# P-selectin-targeted nanocarriers induce active crossing of the blood–brain barrier via caveolin-1-dependent transcytosis

**DOI:** 10.1038/s41563-023-01481-9

**Published:** 2023-03-02

**Authors:** Daniel E. Tylawsky, Hiroto Kiguchi, Jake Vaynshteyn, Jeffrey Gerwin, Janki Shah, Taseen Islam, Jacob A. Boyer, Daniel R. Boué, Matija Snuderl, Matthew B. Greenblatt, Yosi Shamay, G. Praveen Raju, Daniel A. Heller

**Affiliations:** 1 Molecular Pharmacology Program, Memorial Sloan Kettering Cancer Center, New York, NY USA; 2 Department of Pharmacology, Weill Cornell Graduate School of Medical Sciences, New York, NY USA; 3 Department of Pediatrics, Memorial Sloan Kettering Cancer Center, New York, NY USA; 4 Departments of Neurology and Pediatrics, Icahn School of Medicine at Mount Sinai, New York, NY USA; 5 Departments of Pathology & Laboratory Medicine, Nationwide Children’s Hospital and The Ohio State University, Columbus, OH USA; 6 Division of Neuropathology, Department of Pathology, NYU Langone Medical Center, New York, NY USA; 7 Department of Pathology and Laboratory Medicine, Weill Cornell Medicine, & Research Division, Hospital for Special Surgery, New York, NY USA; 8 Faculty of Biomedical Engineering, Technion Israel Institute of Technology, Haifa, Israel; 9 Department of Pediatrics, Weill Cornell Medicine, New York, NY USA

**Keywords:** Nanoparticles, Targeted therapies, Caveolae, Drug delivery

## Abstract

Medulloblastoma is the most common malignant paediatric brain tumour, with ~30% mediated by Sonic hedgehog signalling. Vismodegib-mediated inhibition of the Sonic hedgehog effector Smoothened inhibits tumour growth but causes growth plate fusion at effective doses. Here, we report a nanotherapeutic approach targeting endothelial tumour vasculature to enhance blood–brain barrier crossing. We use fucoidan-based nanocarriers targeting endothelial P-selectin to induce caveolin-1-dependent transcytosis and thus nanocarrier transport into the brain tumour microenvironment in a selective and active manner, the efficiency of which is increased by radiation treatment. In a Sonic hedgehog medulloblastoma animal model, fucoidan-based nanoparticles encapsulating vismodegib exhibit a striking efficacy and marked reduced bone toxicity and drug exposure to healthy brain tissue. Overall, these findings demonstrate a potent strategy for targeted intracranial pharmacodelivery that overcomes the restrictive blood–brain barrier to achieve enhanced tumour-selective penetration and has therapeutic implications for diseases within the central nervous system.

## Abstract (web summary)

Targeting of tumour vasculature endothelial P-selectin promotes caveolin-1-mediated transcytosis for enhanced blood–brain barrier crossing of therapeutic nanoparticles against medulloblastoma.

## Main

Site-directed drug delivery to brain parenchymal tissue is a desirable but elusive goal due to the distinct and highly regulated blood–brain barrier (BBB). The BBB is comprised of a network of specialized endothelial cells, pericytes and astrocytes that prevent material extravasation^1^. Characteristic tight junctions between adjacent brain endothelial cells block paracellular transport, and the passive entry of molecules is constrained to a narrow window of size and lipophilicity. These restrictive physical and functional barriers inhibit drug exposure to intracranial tissues^2^. Hydrophilic small-molecule drugs are routinely excluded from the brain by tight junctions and, while many lipophilic drugs are capable of entry via passive diffusion, penetration into diseased brain tissue is inefficient and typically requires high drug doses, often resulting in dose-limiting systemic toxicity^3^. As such, the integrity of the BBB can considerably impact treatment efficacy. This is well evidenced in medulloblastoma wherein patients in the Sonic hedgehog medulloblastoma subgroup (SHH-MB) suffer worse clinical outcomes due, in part, to an intact BBB limiting the entry of drugs into the brain at therapeutic concentrations^4–6^. In addition, targeted inhibition of the SHH effector Smoothened (SMO) via vismodegib causes premature bone growth plate fusion in paediatric patients, probably as a result of the high doses required for therapeutic efficacy^7,8^.

Given the many limitations for the passage of small molecules across the BBB, nanoparticles have been explored as a vehicle to improve delivery into brain tissues^9^. To date, much of this work has focused on strategies that enhance passive mechanisms of transport for drug-loaded nanoparticles across the BBB. For instance, in diseases that result in a compromised BBB such as glioblastoma, nanostructures have been observed to extravasate through the leaky vasculature to accumulate at tumour sites^10^. Also, researchers have implemented analogous approaches to improve drug delivery past an intact BBB by developing strategies that first disrupt this barrier^11–13^. However, by permitting unregulated passage across the BBB, such approaches not only abrogate the homeostatic functions of the BBB but potentially expose the brain to harmful toxins and pathogens. Alternative approaches for diseases such as SHH subgroup medulloblastoma that retain BBB integrity have utilized nontargeting nanocarriers to extend systemic circulation of small-molecule drugs, with only partial improvement of on-target toxicity profiles at high doses^14^. Importantly, recent work has suggested that passive entry of nanoparticles into solid tumours through gaps between endothelial cells represents a minor mechanism of entry and that up to 97% of transport is through an active process through endothelial cells^15^. However, the molecular mechanism of this active transcellular transport across endothelial barriers has not yet been elucidated and little is known of whether this transendothelial nanoparticle transport occurs at the BBB.

In this study, we investigated active transcellular transport mechanisms to enhance drug delivery across an intact BBB specifically to brain tumour tissue. We and others previously demonstrated targeting of fucoidan-based nanoparticles to P-selectin on activated endothelial cells and found that P-selectin expression on endothelial cells may be enhanced by radiotherapy (RT) to effect greater nanoparticle accumulation in tumour sites^16,17^. Herein, we found that P-selectin facilitates material transendothelial transport across an intact BBB via caveolin-1 (Cav1)-mediated transcytosis. Using a genetic mouse model of SHH-MB with an intact BBB, we found that P-selectin targeting results in active transport in tumour endothelium to enable delivery of fucoidan-based nanoparticles selectively into the tumour microenvironment, which is enhanced by RT. Fucoidan nanoparticles encapsulating the Smoothened inhibitor vismodegib (FiVis) exhibited potent effector inhibition at low drug doses, striking antitumour efficacy and attenuated on-target bone-related toxicities. These findings demonstrate a targeted approach for improving the therapeutic index of vismodegib for SHH-MB and present a potent adjuvant strategy for delivery of drugs to treat brain tumours in combination with standard RT. Furthermore, we report an active mechanism of transendothelial transport that can be exploited to improve drug delivery across activated brain endothelial cells at sites of intracranial disease in conditions with an intact BBB.

## Low-dose irradiation enhances P-selectin on tumour vasculature

**Extended Data Fig. 1:**
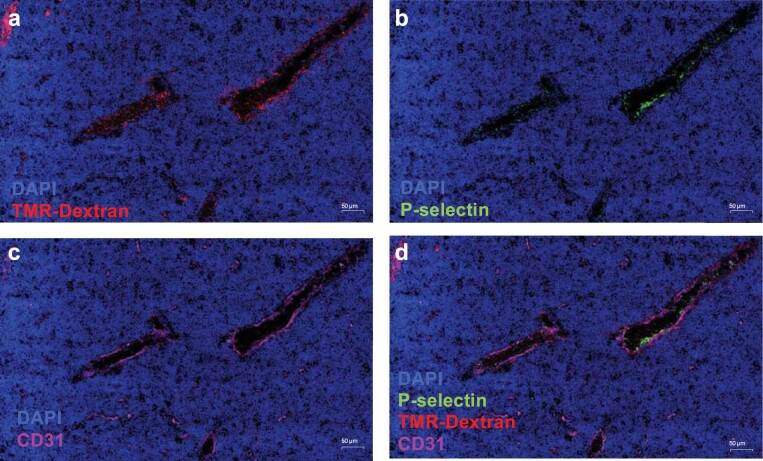
BBB integrity in the SHH-MB GEM model. (**a**–**d**) Immunofluorescence staining of a representative *Ptf1a*^*cre*^*;Ptch1*^*fl/fl*^ SHH-MB tumor two hours after intravenous administration of 70 kDa tetramethylrhodamine (TMR)-dextran. Mice were dosed with 0.25 Gy of ionizing radiation two hours prior to injection of TMR-Dextran. *n* **=** 3 mice. CD31 on endothelium (violet), DAPI nuclear stain (blue), P-selectin (green) and TMR-dextran (red) are shown where indicated.

We first characterized the brain vasculature in a genetically engineered mouse (GEM) *Ptf1a*^*cre/+*^*;Ptch1*^*fl/fl*^ SHH-MB model to investigate BBB integrity. To assess the permeability of the BBB in mice at advanced stages of SHH-MB, symptomatic mice (14 weeks or older) were injected intravenously with tetramethylrhodamine (TMR)-dextran. We observed minimal extravasation of TMR-dextran into parenchymal brain tumour tissue, including following the administration of low-dose ionizing radiation (Extended Data Fig. 1). We conclude that the BBB of these mice appears to remain intact well into advanced tumour stages and thus parallels the physiology of human patients with SHH-MB^6^.

**Fig. 1:**
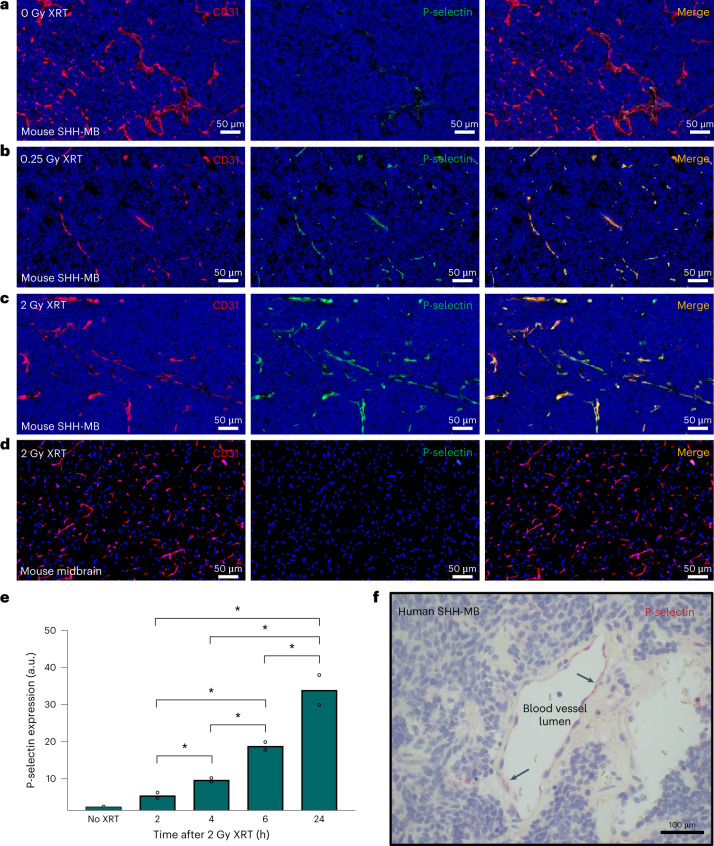
Low-dose irradiation induces P-selectin expression on tumour vasculature in medulloblastoma. **a**–**c**, Immunofluorescence of P-selectin (green) and vasculature (CD31, red) in SHH-MB brain tumour tissues in nonirradiated mice (**a**), 4 h after 0.25 Gy XRT (**b**) and 4 h after 2 Gy XRT (**c**). **d**, Immunofluorescence of adjacent nontumour tissue taken from the midbrain region of whole-body-irradiated, tumour-bearing mice. **e**, Time course of P-selectin expression in SHH-MB tumour tissue following 2 Gy XRT, quantified from immunoblot analysis. *n* = 2 mice per group; *P < 0.05 (two-tailed *t*-test). Data are means ± s.e.m. **f**, Representative human SHH-MB tumour tissue immunostained (red) for P-selectin expression. Haematoxylin counterstaining (blue) indicates MB tumour tissue. Source data

**Extended Data Fig. 2:**
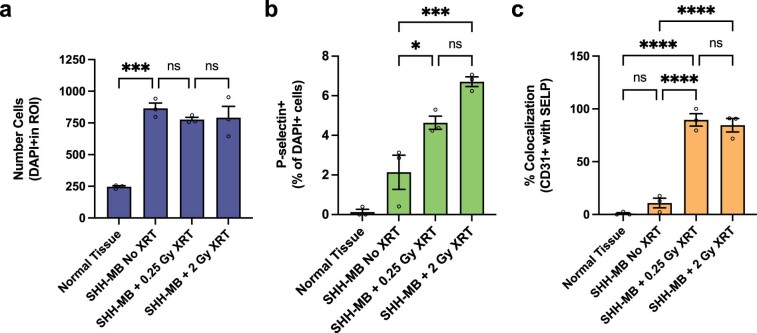
Quantification of immunofluorescence imaging data from Fig. 1a–d. Quantification of (**a**) cell number, (**b**) P-selectin expression, and (**c**) co-localization of P-selectin with CD31 per ROI shown. Data are means ± SEM, *P < 0.05, **P < 0.01, ***P < 0.001, ****P < 0.001, (one-way ANOVA); ns, not significant. Quantification was performed using QuPath bioimage software. Source data

**Extended Data Fig. 3:**
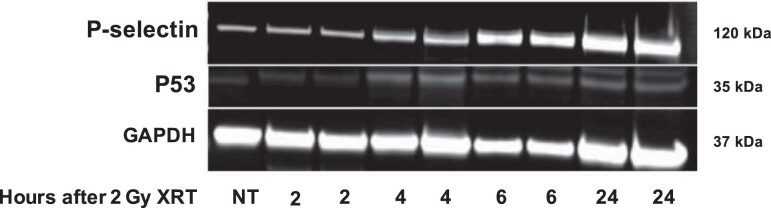
Timecourse of P-selectin expression in SHH-MB following ionizing radiation. Western blot analysis of *Ptf1a*^*cre*^*;Ptch1*^*fl/fl*^ SHH-MB tumors following 2 Gy irradiation. P-selectin protein expression assessed at 2, 4, 6, and 24 hour time points (n = 2 mice at each time point) and untreated control (NT). P53 immunoblotting is used to benchmark exposure to ionizing radiation. GAPDH is used as a loading control. Source data

We next examined P-selectin expression in the SHH-MB tumour microenvironment and the effects of low-dose X-ray irradiation (XRT)^18^. We found that P-selectin is expressed in SHH-MB tumour vasculature in the absence of radiation (Fig. 1a) and that this expression could be further enhanced following a single 2 Gy dose of XRT (Fig. 1c). Notably, the elevation of P-selectin expression following whole-brain irradiation was confined to tumour regions and was not apparent in adjacent, normal brain tissue (Fig. 1d). To assess the potential to mitigate RT-related toxicity, we sought to identify the minimal required dose of irradiation that could still achieve robust induction of endothelial P-selectin expression. We found that P-selectin expression could still be robustly induced following a single dose of 0.25 Gy XRT (Fig. 1b and Extended Data Fig. 2). P-selectin expression was observed to reach substantially elevated levels at approximately 6 h following XRT, and these levels persisted for at least 24 h (Fig. 1e and Extended Data Fig. 3). To confirm the clinical relevance of endothelial P-selectin expression as a potential target molecule, we examined human SHH-MB tumour tissue surgically resected from paediatric patients. Immunohistochemical analysis similarly showed P-selectin expression in tumour-adjacent vasculature (Fig. 1f).

## P-selectin-targeted nanoparticles cross the BBB

**Fig. 2:**
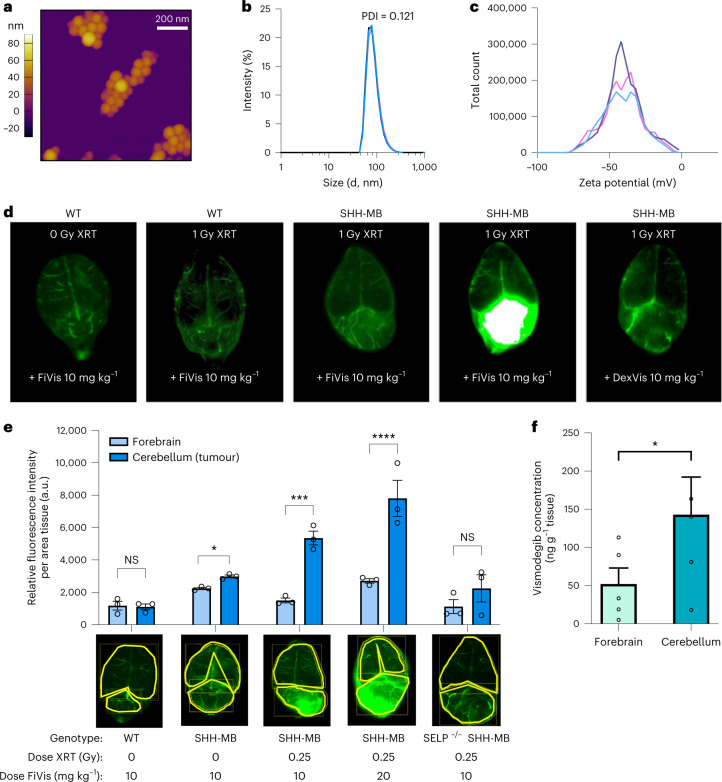
P-selectin-targeted nanoparticles preferentially target SHH-MB tumours following low-dose irradiation. **a**, Atomic force micrograph of FiVis nanoparticles. **b**, DLS data showing average size of FiVis nanoparticles (as quantified by intensity distribution). PDI, polydispersity index; d, diameter. **c**, Zeta potential measurements of FiVis nanoparticles. **d**, Representative near-infrared images (dorsal view) of brains from WT and SHH-MB mice administered FiVis nanoparticles. *n* = 3 mice per group in each of two independent experiments. **e**, Top, near-infrared fluorescence intensities of FiVis nanoparticles in tumour regions (cerebellum) and nontumour regions (forebrain) of WT, SHH-MB and P-selectin (SELP) null SHH-MB mice. Bottom, representative dorsal images of brains, demarcated by cerebellum and forebrain (yellow) for quantification. *n* = 3 mice per group; *P < 0.05, **P < 0.01, ***P < 0.001 (two-tailed *t*-test); NS, not significant. **f**, Liquid chromatography–tandem mass spectrometry quantification of vismodegib in cerebellar and forebrain tissue of mice treated with 0.25 Gy XRT and 10 mg kg^–1^ FiVis. *n* = 5 mice per group; *P < 0.05, *P* = .0424 (paired, two-tailed *t*-test). **e**,**f**, Data are means ± s.e.m. Source data

**Extended Data Fig. 4:**
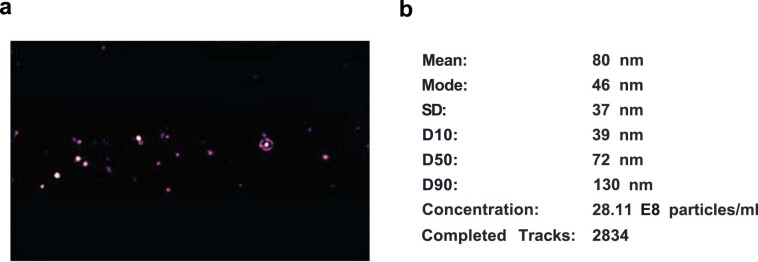
Nanoparticle tracking analysis of FiVis suspension. (**a**) Representative image of FiVis nanoparticles by a NanoSight instrument. (**b**) Summary of results from nanoparticle tracking analysis of FiVis. D10: 10% of particles exhibit a diameter below this value; D50: Median diameter; 50% of particles have a diameter below this value; D90: 90% of particles measured have a diameter below this value. Source data

**Extended Data Fig. 5:**
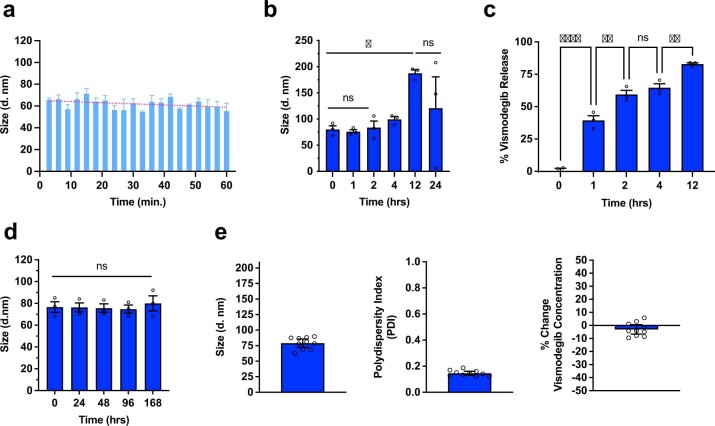
Size and stability of FiVis nanoparticles. **(a)** DLS data showing the average diameter of FiVis nanoparticles after incubating in 25% FBS solution (in PBS) for 1 hour. (**b)** Size and (**c)** drug release profile of FiVis nanoparticles incubated at 37 °C (25% adult bovine serum in PBS). Evaluated by HPLC quantification of vismodegib released into solution over time. **(d)** Size of FiVis nanoparticles measured by DLS, removed from storage at 4 °C before each measurement. Data are means ± SEM. ns, not significant (unpaired t test). Data in (**a-d**) are means ± SEM. *n* = 3 experimental samples per group; *P < 0.05, **P < 0.01, ***P < 0.001, ****P < 0.001 (two-sided t test); ns, not significant. **(e)** Size, PDI, and drug encapsulation of resuspended FiVis nanoparticles after lyophilization. Data are means ± SEM. *n* = 10 experimental samples per group. Source data

To target an SHH pathway modulator to P-selectin, we synthesized nanoparticles encapsulating the Smoothened inhibitor vismodegib. We used a nanoprecipitation process, incorporating the polysaccharide fucoidan, to target P-selectin and a near-infrared dye (IRDye783), to facilitate imaging^16^. The resulting FiVis nanoparticles exhibited an average size of 80 ± 10 nm and were relatively homogeneous as determined by atomic force microscopy (Fig. 2a), dynamic light scattering (DLS) (Fig. 2b,c) and nanoparticle tracking analysis (Extended Data Fig. 4). FiVis nanoparticles were also tested for their stability in serum and exhibited effective drug encapsulation for up to 12 h as determined by quantification of free drug released into the serum mixture, which would be indicative of nanoparticle disassembly (Extended Data Figs. 3b and 5a–c).

**Extended Data Fig. 6:**
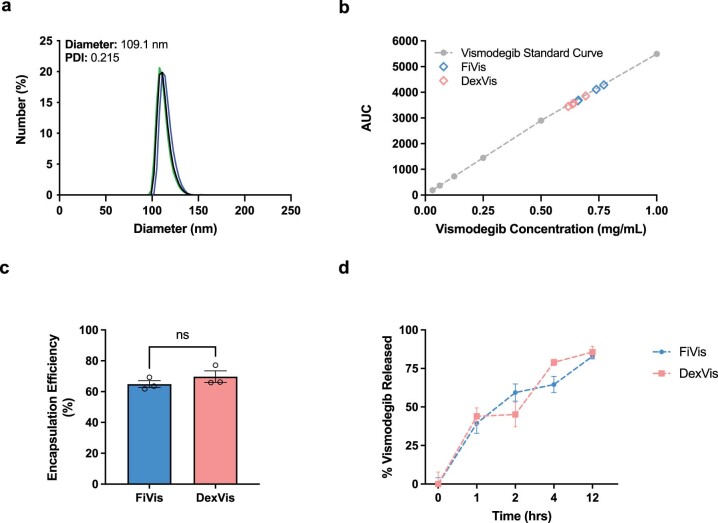
Characterization of dextran sulfate/vismodegib nanoparticles. (**a**) Dynamic light scattering characterization of DexVis nanoparticles. (**b**) HPLC quantification of vismodegib in DexVis and FiVis nanoparticle formulations from three preparations, and a standard curve of vismodegib. (**c**) Encapsulation efficiencies of FiVis and DexVis nanoparticles. Data are means ± SEM. *n* = 3 samples from independent experiments; *ns*, not significant (two-tailed t test). (**d**) Drug release profile of FiVis and DexVis nanoparticles incubated at 37 °C (25% adult bovine serum in PBS). Evaluated by HPLC quantification of vismodegib released into solution over time. Data are means ± SEM. *n* = 3 samples from independent experiments. Source data

**Extended Data Fig. 7:**
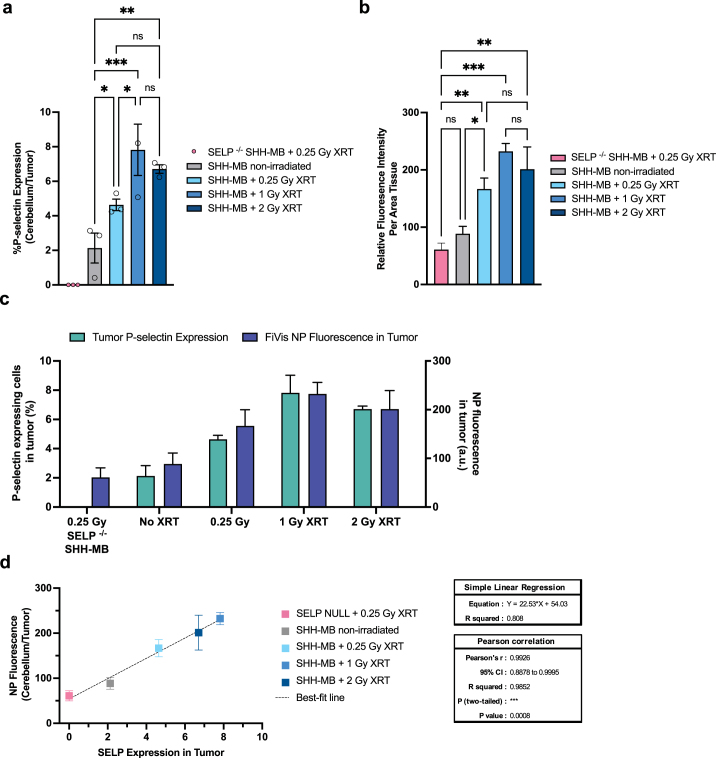
Quantification of P-selectin expression and FiVis targeting to SHH-MB tumors. (**a**) P-selectin expression in SHH-MB tumor tissue upon treatment with various doses of ionizing radiation. (**b**) Near-IR fluorescence intensities of FiVis nanoparticles in tumor regions (cerebellum) of SHH-MB mice treated with FiVis nanoparticles (10 mg/kg) and various doses of ionizing radiation. Data in (**a, b**) are means ± SEM. n = 3 mice per group; *P < 0.05, **P < 0.01, ***P < 0.001, (one-way ANOVA); ns, not significant. (**c**) Dual axis representation of P-selectin expression and FiVis nanoparticle fluorescence in SHH-MB tumor at corresponding irradiation doses. (**d**) Correlation analysis comparing P-selectin expression and uptake of FiVis nanoparticles observed in tumor. Source data

**Extended Data Fig. 8:**
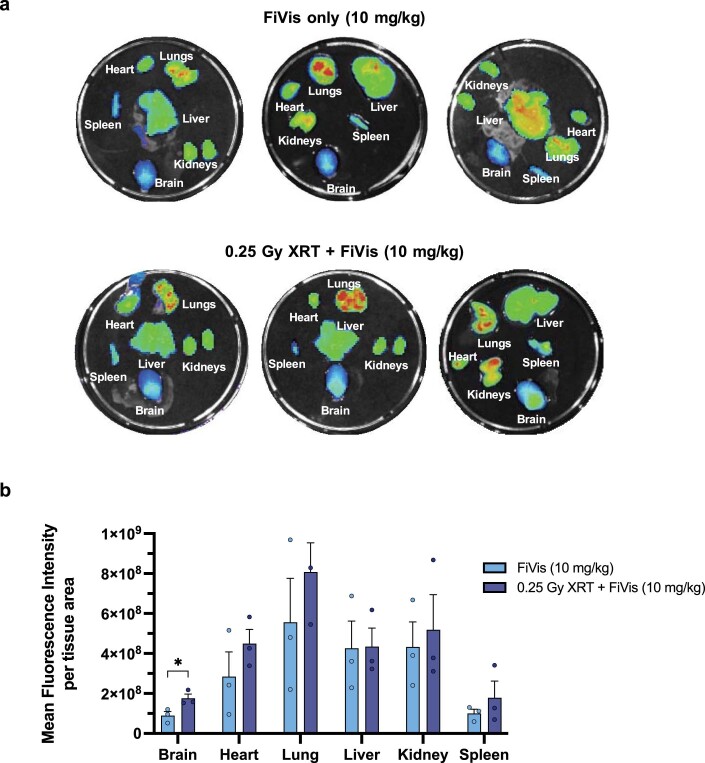
Biodistribution of FiVis nanoparticles in SHH-MB mice. (**a)** Images acquired by IVIS® Spectrum in vivo imaging system showing FiVis biodistribution. SHH-MB mice were treated with either FiVis nanoparticles or a combination of irradiation and nanoparticles. Fluorescence intensities correspond to detection of the IR dye present in these nanoparticles. (**b)** Mean fluorescence intensity per tissue area was quantified for brain, heart, lung, liver, kidneys, and spleen. Data are means ± SEM. *n* = 3 mice per group. For each organ, the two indicated treatment groups were compared by unpaired, two-tailed *t*-test; significant differences are indicated (*P < 0.05, *P* = *0.0393*), otherwise differences are not significant. Source data

**Extended Data Fig. 9:**
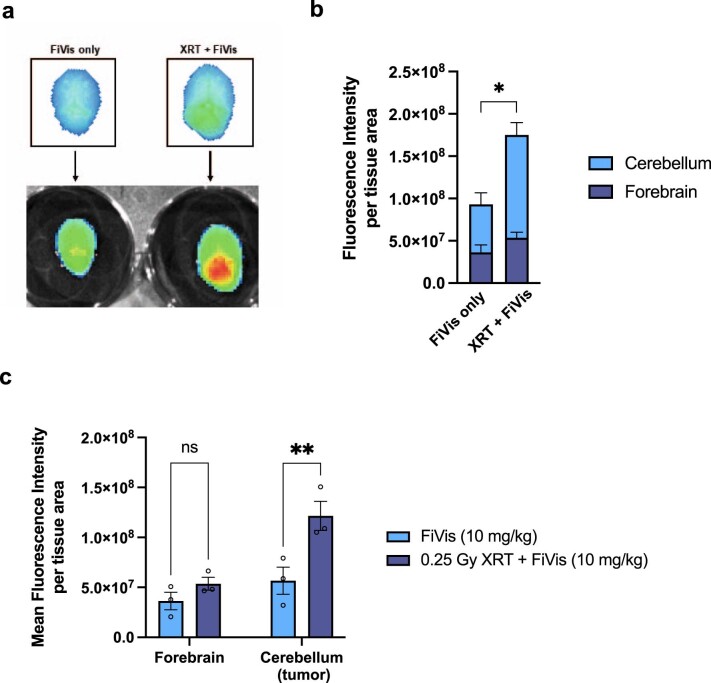
Imaging and quantification of FiVis biodistribution to the brain. (**a**) Side-by-side imaging of nanoparticle fluorescence in SHH-MB GEM mouse brains resected from mice treated with either FiVis nanoparticles only or a combination of irradiation and nanoparticles (XRT + FiVis). (**b, c**) Quantification of FiVis nanoparticle fluorescence intensity in cerebellum (tumor tissue) compared to forebrain (normal tissue) in non-irradiated and 0.25 Gy irradiated mice. Images acquired by IVIS® Spectrum in vivo imaging system. For (**a-c**), groups treated with nanoparticles only were dosed with 10 mg/kg FiVis, and groups treated with both irradiation and nanoparticles were dosed with 0.25 Gy XRT and 10 mg/kg FiVis. Data in (**b, c**) are means ± SEM. n = 3 mice per group; *P < 0.05, **P < 0.01, (two-way ANOVA); ns, not significant. Source data

**Extended Data Fig. 10:**
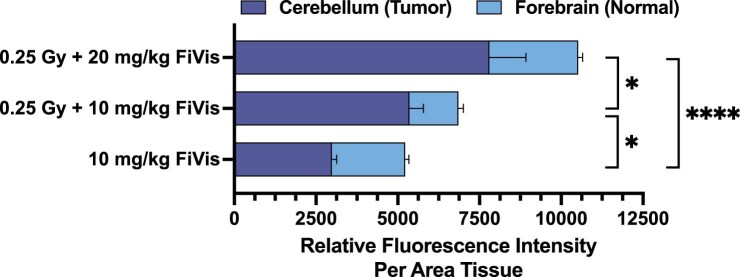
Dose response of FiVis nanoparticle uptake in tumors. Quantification of nanoparticle fluorescence localized at the cerebellum or forebrain of SHH-MB mice treated with 10 mg/kg or 20 mg/kg doses of FiVis and 0.25 Gy XRT. Data are means ± SEM. n = 3 mice per group; *P < 0.05, ****P < 0.0001, (two-way ANOVA). Source data

We sought to assess nanoparticle localization to SHH-MB tumours and the effects of RT. In nonirradiated mice, treatment with FiVis nanoparticles resulted in a slight increase in targeting to the cerebellar tumour compared with adjacent normal forebrain tissue (Fig. 2d). However, in mice pretreated with irradiation (1 Gy) we observed pronounced tumour accumulation of FiVis emission in contrast to nontumour-bearing WT mice, where it remained apparently intravascular. We also synthesized control dextran sulfate-based vismodegib (DexVis) nanoparticles that do not exhibit affinity to P-selectin (Extended Data Fig. 6)^19^. These nanoparticles, exhibiting size and drug encapsulation characteristics similar to FiVis, did not exhibit comparable tumour localization, even following irradiation (Fig. 2d). Upon varying the dose of radiation, we found that both P-selectin enhancement and tumour accumulation of FiVis NPs largely increased with XRT dose (Extended Data Fig. 7). Treatment with 0.25 Gy XRT resulted in upwards of a threefold increase in FiVis nanoparticle localization at tumour sites as compared with normal adjacent forebrain tissue (Fig. 2e and Extended Data Figs. 8 and 9), and the signal increased with nanoparticle dose (Extended Data Fig. 10). Localization of FiVis nanoparticles to tumours was abrogated in P-selectin knockout (KO) SHH-MB mice (SELP null SHH-MB, *Ptf1a*^*cre/+*^*; Ptch1*^*fl/fl*^*; Selp*^*−/−*^), further confirming the importance of P-selectin for BBB penetration. To assess the fate of the nanoparticle drug cargo, we measured vismodegib by liquid chromatography–mass spectrometry (LC–MS). We found substantially preferential accumulation of the drug within cerebellar tumour regions as compared with normal adjacent forebrain regions in mice treated with FiVis and 0.25 Gy XRT (Fig. 2f).

## Caveolae mediate active transport of particles across the BBB

**Fig. 3:**
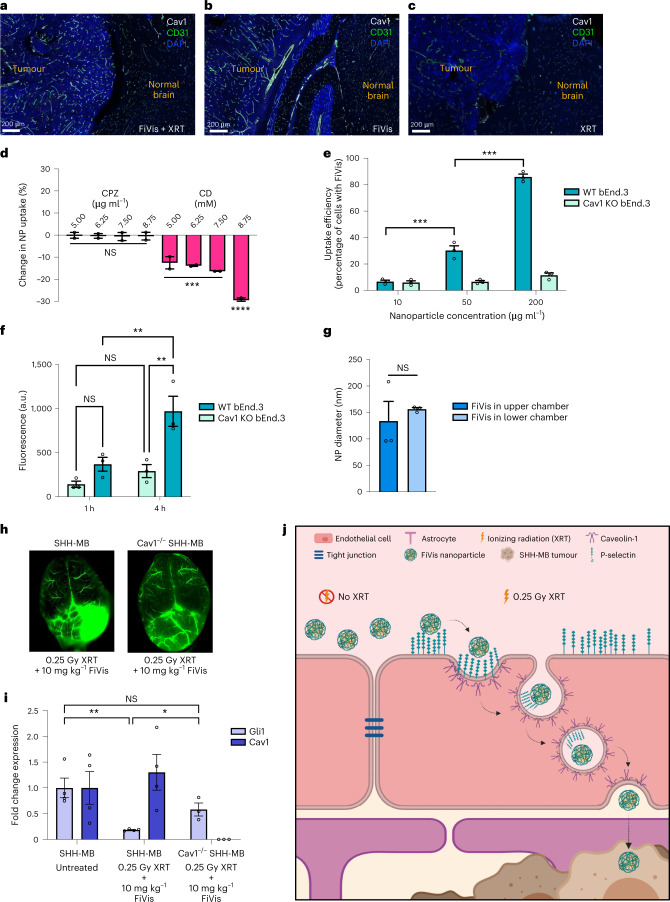
Passage of FiVis nanoparticles across the BBB is mediated by Cav1. **a**–**c**, Immunofluorescence of Cav1 (white) and CD31 (green) in advanced SHH-MB mouse tumours following treatment with 0.25 Gy XRT + FiVis (**a**), FiVis only (**b**) or XRT only (**c**). **d**, Uptake of FiVis nanoparticles (NP) into bEnd.3 cells following pretreatment with pharmacological inhibitors of endocytosis pathways, as measured by flow cytometry. Uptake was compared to that in a group of cells administered nanoparticles but no inhibitor. Data are means ± s.e.m. *n* = 3 biologically independent samples; ***P < 0.001, ****P < 0.001 (one-way ANOVA). **e**, Uptake of FiVis nanoparticles at indicated doses in Cav1 WT bEnd.3 cells compared with that in homozygous Cav1KO bEnd.3 cells. Data are means ± s.e.m. *n* = 3 biologically independent samples; ***P < 0.001, ****P < 0.001 (one-way ANOVA). **f**, Quantification of FiVis transcytosis across a monolayer of WT or Cav1KO bEnd.3 cells at indicated incubation times using a transwell assay. Data are means ± s.e.m. *n* = 3 biologically independent samples; *P < 0.05 (one-way ANOVA). **g**, DLS assessment of FiVis nanoparticle size in apical and basolateral chambers of a transwell assay across a bEnd.3 cell monolayer. Data are means ± s.e.m. *n* = 3 biologically independent samples. **h**, Near-infared imaging of FiVis nanoparticles at 6 h following XRT and nanoparticle administration at indicated doses in advanced-stage Cav1 WT SHH-MB or Cav1 null SHH-MB tumours. Representative images, *n* = 3 mice per group. **i**, Quantitative real-time PCR analysis of Gli1 target inhibition in advanced-stage Cav1 WT SHH-MB and homozygous Cav1 null SHH-MB tumours following indicated XRT and FiVis treatments. RT–qPCR analysis for Cav1 is shown to indicate Cav1 status in WT and Cav1KO SHH-MB tumours. Data are means ± s.e.m. *n* = 3 mice per group; **P < 0.01, ***P < 0.001 (two-sided *t*-test). **j**, Schematic of proposed mechanism for FiVis nanoparticle passage across the BBB. Source data

We next investigated the mechanism of material transport responsible for delivery of FiVis across the BBB. We observed from immunohistological analysis that Cav1 expression was enhanced in endothelial cells within SHH-MB tumour regions when mice were treated with FiVis nanoparticles (Fig. 3a–c and quantified in Supplementary Fig. 1). We also found that FiVis enhanced Cav1 expression in endothelial cells in vitro (Supplementary Fig. 2a). This finding prompted us to evaluate endocytosis pathways that might potentially mediate this nanoparticle uptake. For pharmacological inhibition of either caveolin- or clathrin-dependent endocytosis, we treated bEnd.3 cells with either methyl-ß-cyclodextrin (CD; an inhibitor of caveolae-dependent endocytosis) or chlorpromazine (CPZ; an inhibitor of clathrin-mediated endocytosis), respectively^20^. While treatment with CPZ did not affect nanoparticle uptake in bEnd.3 cells, groups treated with CD showed significantly reduced FiVis uptake in a dose-dependent manner (Fig. 3d–f). In the presence of 0.25 Gy XRT, which significantly enhanced P-selectin expression on bEnd.3 cells and also resulted in enhanced uptake of FiVis nanoparticles into bEnd.3 cells, we saw similarly reduced uptake following the introduction of CD (Supplementary Fig. 3). We observed a similar trend following inhibition of these pathways via short-hairpin RNA-mediated knockdown of Cav1 and clathrin heavy-chain (CLTC) genes (Supplementary Fig. 4). These results suggest that FiVis entry into murine brain endothelial cells was mediated by caveolin-dependent endocytosis.

To further investigate caveolin-mediated transcytosis, we assessed nanoparticle uptake using Cav1 KO (Cav1KO) bEnd.3 cells (Supplementary Fig. 2b). We observed that FiVis uptake into Cav1KO cells was significantly less than that of wild-type (WT) cells (Fig. 3e). We next sought to determine whether Cav1 also contributes to the transcytosis of FiVis nanoparticles across brain endothelial cells. Cav1KO and WT bEnd.3 cells were cultured on porous transwell inserts for direct assessment of transcellular transport. Confluent cell monolayers were evaluated for integrity by transendothelial electrical resistance (TEER) and paracellular permeability (Supplementary Fig. 5). FiVis nanoparticles introduced to the upper chamber of the insert were quantified by fluorescence in the bottom chamber over the course of a 4 h incubation. The passage of FiVis across the cells and into the bottom chamber was significantly restricted in Cav1KO compared with WT cells (Fig. 3f and Supplementary Fig. 6). Of note, DLS data showed FiVis nanoparticle-sized objects in the bottom chamber after incubation, suggesting that intact particles had passaged across bEnd.3 cells (Fig. 3g), consistent with a transcytosis mechanism.

To evaluate the role of Cav1 in material transport across the BBB in vivo, we bred the Cav1 null allele onto our SHH-MB GEM model to generate homozygous Cav1 null SHH-MB mice (*Ptf1a*^*cre/+*^*; Ptch1*^*fl/fl*^*; Cav1*^*−/−*^). These Cav1 null SHH-MB tumour mice showed latency and penetrance similar to those in Cav1 WT SHH-MB mice, with resultant tumours having similar histological features and levels of P-selectin expression (Supplementary Fig. 7). We administered FiVis to these Cav1KO SHH-MB mice following low-dose XRT and observed that nanoparticles localized to tumour vasculature (Fig. 3h). We surmise that the nanoparticles bound to endothelial P-selectin in this case but that they could not extravasate into the brain tumour parenchyma. Furthermore, SHH pathway target inhibition was considerably diminished in Cav1 null SHH-MB mice as evident by the abrogation of Gli1 target inhibition following treatment with FiVis nanoparticles compared with that in Cav1 WT SHH-MB mice (Fig. 3i). These results support a role for Cav1 in this active nanoparticle transport process across the BBB. A proposed transport model for fucoidan-based nanoparticle drug delivery across the BBB is shown in Fig. 3j.

For live cell imaging and electron microscopy studies, we synthesized gold-core, fucoidan-bound nanoparticles (FiGNPs). FiGNPs exhibited size and zeta potential characteristics similar to those of FiVis nanoparticles (Supplementary Fig. 8). We investigated the dependence on P-selectin and Cav1 on FiGNP uptake in bEnd.3 endothelial cells (Supplementary Fig. 9), finding that it was markedly increased by upregulation of both. We also assessed Cav1 dependence on transcytosis using a transwell assay (Supplementary Fig. 10), finding strong inhibition following *CAV1* KO, similar to the results with FiVis nanoparticles. To visualize the interplay of fucoidan nanoparticles with key proteins of interest at spatiotemporal resolution, we conducted live cell imaging studies with FiGNPs. We modified bEnd.3 brain endothelial cells to stably express both green fluorescent protein (GFP)-tagged P-selectin and mCherry-tagged Cav1 (Supplementary Figs. 11 and 12). Confocal live cell imaging studies showed FiGNP engagement with P-selectin and subsequent uptake in Cav1-associated vesicles (Supplementary Figs. 13 and 14 and Supplementary Video 1).

We conducted transmission electron microscopy imaging of cerebellar tumour tissue from SHH-MB mice treated with FiGNPs. We first confirmed that FiGNPs administered to irradiated (1 Gy) SHH-MB mice exhibit similar extravasation within the cerebellum (Supplementary Fig. 15). On imaging tumour-proximal endothelium in osmium tetroxide-stained tissue sections via transmission electron microscopy, we found endosomal uptake of FiGNPs. The vesicles harbouring FiGNPs appeared to be both caveolae and lysosomes. These data suggest that FiGNPs are taken up into vesicles/caveolae by endothelial cells that form the BBB (Supplementary Fig. 16).

## Vismodegib efficacy is enhanced by targeted NP delivery

**Fig. 4:**
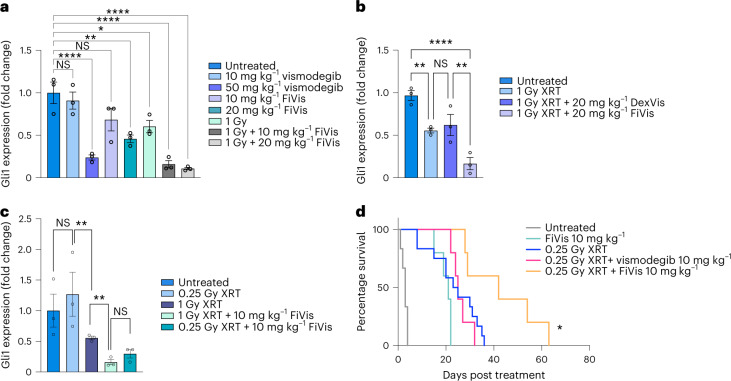
FiVis nanoparticles synergize with low-dose irradiation to enhance Gli1 target inhibition and survival in SHH-MB. **a**–**c**, Quantitative real-time PCR analysis of Gli1 target inhibition in advanced-stage SHH-MB tumours following indicated treatments. **a**, RT–qPCR analysis of Gli1 expression comparing free vismodegib with FiVis at indicated doses, either alone or in combination with ionizing radiation. **b**, RT–qPCR analysis of Gli1 expression comparing treatment of P-selectin-targeting FiVis with control nontargeting DexVis at indicated doses in combination with ionizing radiation. **c**, RT–qPCR analysis of Gli1 expression following FiVis treatment at indicated dose with very-low-dose (0.25 Gy) ionizing radiation. **a**–**c**, Data are means ± s.e.m. *n* = 3 mice per group; *P < 0.05, **P < 0.01, ***P < 0.001, ****P < 0.001 (two-sided *t*-test). **d**, Kaplan–Meier survival analysis of advanced-stage SHH-MB mice treated with either fractionated 0.25 Gy XRT, low-dose FiVis, free vismodegib or combinations thereof at indicated doses. Grey, survival of untreated SHH-MB mice. XRT doses of 0.25 Gy alone or in combination with respective drugs were given every other day in eight doses. *P = 0.0198 (log-rank, Mantel–Cox) for XRT + FiVis (orange) compared with XRT + vismodegib (pink). *P* not significant (log-rank, Mantel–Cox) when XRT + vismodegib (pink) compared with XRT alone (blue). Source data

We next proceeded to test the therapeutic efficacy of FiVis nanoparticle treatment in SHH-MB mice. We used reverse transcription-quantitative polymerase chain reaction (RT-qPCR) to measure the expression of Gli1, a downstream effector of SHH pathway activation^21^. When combined with 1 Gy XRT, FiVis treatment resulted in Gli1 target inhibition in a dose-dependent manner (~80% inhibition with FiVis 10 mg kg^–1^ and ~90% inhibition with FiVis 20 mg kg^–1^) (Fig. 4a). In contrast, SHH-MB mouse models typically require treatment with free drug vismodegib doses upwards of 50 mg kg^–1^ to achieve comparable levels of Gli1 inhibition^21^. Treatment with control DexVis nanoparticles in combination with XRT did not result in Gli1 inhibition as compared with XRT alone (Fig. 4b). Furthermore, the improved vismodegib efficacy conferred by a combination of XRT and FiVis nanoparticle treatment was sustained even with a very low (0.25 Gy) XRT dose, which alone had only a marginal effect on both Gli1 levels and apoptosis in tumours (Fig. 4c and Supplementary Fig. 17).

Using these Gli1 inhibition data to inform nanoparticle drug dosing in a survival study, we tested the efficacy of low-dose XRT and FiVis in treatment of mice with advanced-stage SHH-MB. Because higher doses of ionizing radiation did not confer a substantial survival benefit, 0.25 Gy XRT was selected for subsequent survival studies (Supplementary Fig. 18). Although treatment with 10 mg kg^–1^ FiVis considerably prolonged survival on its own, when combined with 0.25 Gy XRT, FiVis treatment at 10 mg kg^–1^ further extended survival by more than twofold (Fig. 4d). We did not observe enhanced mouse survival by administration of free vismodegib at 10 mg kg^–1^ following 0.25 Gy XRT, suggesting that the efficacy observed with P-selectin-targeted FiVis nanoparticles was probably not mediated by any potential BBB leakiness induced by this low level of ionizing radiation. To confer a survival benefit, free vismodegib must be given at much higher doses; we thus administered vismodegib at a considerably elevated (50 mg kg^–1^) dose to determine comparable efficacy (Supplementary Fig. 19). Others have administered vismodegib as high as twice daily at 92 mg kg^–1^ to determine substantial antitumour effects^21^.

## Nanoparticles abrogate vismodegib toxicities

**Fig. 5:**
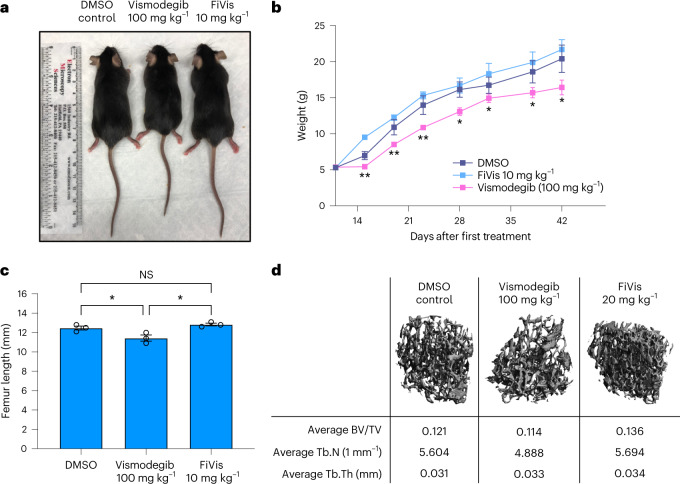
FiVis nanoparticles abrogate vismodegib treatment-related bone toxicity in juvenile mice. **a**, Body length of mice at 6 weeks of age following 2 day treatment at age 10 days (P10) with vismodegib (free Vis), FiVis or DMSO vehicle at indicated doses. Representative image; *n* = 3 mice per group. **b**, Mouse body weight over 6 weeks following 2 day treatment of P10 mice with vismodegib (100 mg kg^–1^), FiVis nanoparticles (10 mg kg^–1^) or DMSO. *n* = 3 mice per group. **c**, Femur length of P10 mice at 6 weeks following 2 day treatment with vismodegib or FiVis nanoparticles. *n* = 3 mice per group; *P < 0.05 (two-sided *t*-test). Data are means ± s.e.m. **d**, Representative microcomputed tomography three-dimensional reconstruction images of trabecular bone in the distal femoral metaphysis taken from mice treated with DMSO control, FiVis nanoparticles (20 mg kg^–1^) or free vismodegib (100 mg kg^–1^). BV/TV, trabecular bone volume/total volume; Tb.N, trabecular number; Tb.Th, trabecular thickness. Source data

We investigated the issue of general and bone toxicities that have been observed in preclinical studies and in children treated with vismodegib^8,22,23^. We found that even short-term treatment of mice aged 10 days (P10) with high doses of vismodegib caused obvious growth stunting (Fig. 5a). Notably, these growth defects did not occur in young mice treated with FiVis at 10 mg kg^–1^, the dose used in our efficacy studies. At a free vismodegib dose of 100 mg kg^–1^, mice exhibited growth restriction while the weights of FiVis-treated mice paralleled those of vehicle-treated control mice (Fig. 5b). Closer examination of bone tissue revealed pronounced abnormalities in femur length (Fig. 5c) and trabecular bone number, using microcomputed tomography (Fig. 5d). Doses of FiVis at 10 or 20 mg kg^–1^ did not result in noticeable toxicities.

## Outlook

The highly specialized endothelial cells that comprise the BBB act as a physiological barrier that regulates the entry of molecules into the central nervous system. While this is favourable for maintaining homeostasis under normal conditions, it is an impediment to effective drug delivery for the treatment of diseases that manifest in parenchymal brain tissue. We report one way to overcome this pervasive challenge, by stimulating the active transport machinery of brain endothelial cells. Here, we found that the targeting of P-selectin on tumour vasculature facilitates Cav1-dependent transendothelial transport and enhances drug delivery across an intact BBB. We investigated this phenomenon using a combination of nanoengineering and genetic approaches. We found that nanoparticles with affinity to P-selectin, effected by a fucoidan polysaccharide coating, can cross the BBB via transendothelial transport through brain endothelial cells in a GEM model with an intact BBB, and that this passage is P-selectin dependent. In addition, using both pharmacological inhibition and genetic KO, we found that the cellular entry and transendothelial BBB transport of P-selectin-targeted materials to SHH-MB tumour tissue is Cav1 dependent and is not likely to occur by passive passage through interendothelial spaces, as suggested by recent studies^15^. This mechanism of transport potentially enables the delivery of nanoparticles and other large cargoes across the BBB without impacting the structural integrity of the neurovascular unit.

This study investigates a specific receptor–ligand interaction to facilitate targeted and controlled delivery of nanoparticle-encapsulated therapeutic agents across the BBB. Previous work by others supports a role for P-selectin-mediated engagement and Cav1 interaction. Notably, activated endothelial cells exhibit substantial colocalization of P-selectin and Cav1, a primary constituent of caveolae^24^. Our data support the notion that the tendency for P-selectin to partition into caveolae may effectively prime P-selectin-bound FiVis nanoparticles for uptake by caveolae-mediated endocytosis and transcytosis across the BBB. Although beyond the scope of this current study, it is currently not known whether engagement of fucoidan nanoparticles with P-selectin activates a signalling pathway to promote Cav1-dependent cellular entry of nanoparticles or whether it is facilitated by more direct mechanical forces. Along these lines, previous work has shown that PSGL-1 binding to P-selectin on endothelial cells involves intracellular PI3K and Src kinases within leucocytes; however, little is known about the related signalling pathways within endothelial cells following P-selectin engagement^25^. Furthermore, recent work has also shown that CD44 and/or spectrin cytoskeletal networks on endothelial cells can restrict selectin mobility to maintain apical density and clustering, thus allowing for leucocyte rolling through PSGL-1 (ref. ^24^). Interestingly, activated endothelial cells following tumour necrosis factor-alpha stimulation also resulted in apical distribution and restricted mobility of caveolae with subsequent colocalization of caveolae with P-selectin. Future studies will be important in distinguishing whether fucoidan nanoparticle engagement with P-selectin on activated endothelial cells results in involvement of either endogenous signalling pathways or alterations in CD44 and/or spectrin cytoskeletal networks for subsequent cellular entry.

The potential clinical applications for this approach are assessed herein. Fucoidan-encapsulated vismodegib was used in this work as proof of principle, and to assess the potential to improve the therapeutic index of a precision therapeutic with a known on-target toxicity. However, many other drugs have been encapsulated in fucoidan-based carriers and other P-selectin-targeted vehicles^10,16,17,26^. Potentially important payloads include drug combinations that can address tumour heterogeneity and treatment resistance mechanisms. Such combinations often result in additive, dose-limiting toxicities. The increased therapeutic indices observed with the P-selectin nanotargeting approach may allow tumour cell autonomous and nonautonomous combination drug treatment strategies to facilitate tolerability in patients. Further clinical applicability of this P-selectin-targeting approach probably extends beyond primary medulloblastoma to other intracranial tumours^10,17^ and metastatic disease^16^. In addition, several central nervous system disorders, including multiple sclerosis^27,28^, ischaemic stroke^29^ and focal epilepsy^30^, have been shown to upregulate restricted endothelial P-selectin expression at sites of disease exacerbation where leucocyte trafficking plays a role in disease pathogenesis. These indications may provide additional opportunities for the targeted delivery of therapeutic agents specifically to sites of intracranial disease, to enhance efficacy while minimizing neurotoxicity and systemic toxicities. We anticipate that the continued investigation and development of methods that harness and improve the transport of materials across the BBB and other endothelial barriers will be instrumental in improving the efficacy of many classes of approved and experimental therapeutics.

## Methods

### Preparation of vismodegib nanoparticles

Fucoidan-encapsulated vismodegib nanoparticles were prepared by a nanoprecipitation method adopted from previous work by our group^16,17^. In a microcentrifuge tube the aqueous phase was prepared by combining the following solutions: 400 μl of fucoidan (15 mg ml^–1^), 50 μl of IR-783 (2 mg ml^–1^), 50 μl of IR-820 (2 mg ml^–1^) and 100 μl of 0.01 mM sodium bicarbonate. While gently vortexing this mixture, 50 μl of vismodegib (20 mg ml^–1^, dissolved in DMSO) was added dropwise. Following complete addition of the organic phase, vortexing was ceased and the resultant semiopaque emulsion centrifuged for 15 min at 30,000*g*. Pellets were resuspended in 200 μl of deionized water and further diluted as necessary, based on the vismodegib concentration of the mixture. Working stocks of FiVis used for in vivo and in vitro experiments were prepared at a concentration 2.5 mg of vismodegib per 1.0 ml of nanoparticle mixture.

### Preparation of FiGNPs

Fucoidan extracted from *Fucus vesiculosus* (no. F5631), gold(III) chloride trihydrate (no. 520918), Pluronic F-127 (no. P2443) and IR-780 iodide dye (no. 425311) were each purchased from Sigma. Briefly, 0.005 g of fucoidan was added to a 10 ml aqueous solution of 1 × 10^−4^ M HAuCl_4_·3H_2_O and the solution was heated to 80 °C with mixing by a magnetic stir bar. After reaching, 80 °C, the solution was mixed for an additional 10 min. During this time, the formation of FiGNPs was indicated by a transition in the colour of the solution from a bright yellow (at the onset of the reaction) to a dark, ruby red (after 10 min of mixing at 80 °C). The solution was then removed from the heat; to better stabilize the colloidal suspension of GNPs, these were incubated in a Pluronic F-127 solution (4 mM final concentration) for 10 min. Next, to load an infrared fluorescent dye onto Pluronic-stabilized FiGNPs, IR-780 iodide was added at a final concentration of 10^−5^ M (0.01 mM) and this solution was mixed at room temperature for 2 h. The resultant colloidal suspension was centrifuged at 30,000*g* for 15 min to collect FiGNPs. After careful removal of the supernatant, the pelleted FiGNPs were washed by redispersing the pellet in deionized water and performing a further centrifugation step.

### Nanoparticle characterization

The concentration of vismodegib in the nanoparticle suspension was quantified using high-performance liquid chromatography (HPLC). Before analysis, nanoparticles were diluted 1:10 in deionized water. An aliquot of this dilution was then mixed with acetonitrile at a ratio of 1:4 to extract vismodegib from the nanoparticles. Samples were then analysed on an Agilent 1260 Infinity II HPLC system with an InfinityLab Poroshell 120 EC-C18, 4.6 × 75 mm^2^, 2.7 µm analytical LC column. The mobile phase comprised acetonitrile and/or deionized water, each containing 0.1% trifluoroacetic acid. Chromatographic separation was achieved by gradient elution with acetonitrile (0–95%) at a flow rate of 1 ml min^–1^. A single peak corresponding to vismodegib was characteristically observed with absorbance at 260 nm and a retention time of 3.04 min. The size and zeta potential of nanoparticles were determined using dynamic and electrophoretic light-scattering measurements acquired with a Malvern Zetasizer Nano ZS.

### Flow cytometry

Murine brain endothelial (bEnd.3) cells were plated in a 12-well plate at a density of 150,000 per well in 1 ml of medium (DMEM, 10% fetal bovine serum (FBS), 1% penicillin/streptomycin). Once confluent, cells in treatment groups receiving ionizing radiation were exposed to 0.25 Gy XRT. After 1 h cells were collected, transferred to microcentrifuge tubes and fixed on ice using 2% paraformaldehyde. Fixed cells were washed twice with PBS and then resuspended in 100 μl of fluorescent activated cell sorter (FACS) buffer (PBS with 2% FBS). Cells were stained with 2 μl of anti-P-selectin antibody (Biolegend, no. 148310) and incubated at room temperature for 30 min. Cells were then washed twice with PBS, resuspended in 300 μl of FACS buffer and transferred to FACS tubes for analysis. Data were collected on a BD LSR II flow cytometer and analysed using either FCS Express Software (v.7.06) or FlowJo (v.10.6.1). To quantify the effects of endocytosis inhibitors on nanoparticle uptake, bEnd.3 cells were plated in a 12-well plate at a density of 150,000 per well in 1 ml of medium. On reaching confluency, cells were treated with medium containing chlorpromazine (5.00, 6.25, 7.50, 8.75 μg ml^–1^), methyl-β-cyclodextrin (5.00, 6.25, 7.50, 8.75 mM) or regular medium. After 8 h, cells were washed with PBS and treated with nanoparticles (1:100 dilution of FiVis in complete DMEM medium). Cells were incubated with nanoparticles for 30 min at 37 °C. Afterwards, cells were washed twice with PBS and resuspended in freshly prepared FACS buffer containing propidium iodide as a viability stain. Data were collected on a BD LSR II flow cytometer using the APC-Cy7 channel (excitation with a 633 nm red laser and detection with a 780/60 nm bandpass filter) to detect fluorescent signal from the infrared dyes within the nanoparticles. Data were analysed using FCS Express Software.

### Fluorescence microscopy

Similar to the nanoparticle uptake experiments using flow cytometry as a readout, bEnd.3 cells were plated in a 12-well plate at a density of 150,000 per well in 1 ml of medium. As above, once the cells were confluent they were treated with medium containing chlorpromazine (5.00, 6.25, 7.50, 8.75 μg ml^–1^), methyl-β-cyclodextrin (5.00, 6.25, 7.50, 8.75 mM) or regular medium. After 8 h cells were washed with PBS and treated with nanoparticles (1:100 dilution of FiVis in complete DMEM medium). Cells were then incubated with nanoparticles for 30 min at 37 °C. After incubation with nanoparticles, cells were washed with Hanks’ buffered salt solution (HBSS) and then stained with an HBSS solution containing both a membrane dye (CellMask Green, diluted 1:1,000) and a nuclear dye (Hoescht 33342, diluted 1:10,000). Following a 15 min incubation at 37 °C, cells were washed twice more with HBSS. Images were taken using an Olympus IX51 fluorescence microscope equipped with XM10IR Olympus camera and an X-Cite Xenon lamp. ImageJ software was used to process the data to create overlays of images taken from different channels.

### Fluorescent protein expression

GFP-tagged P-selectin was stably introduced into bEnd.3 cells via lentiviral transduction. To produce lentivirus, transfer plasmid pLV-mSELP-GFPSpark (Sino Biological, no. MG50737-ACGLN), along with helper plasmids psPAX2 (Addgene plasmid no. 12260) and pMD2.G (Addgene plasmid no. 12259), were transfected into LentiX (Takara Bio) cells at 70% confluence using the transfection agent Lipofectamine 3000 (Invitrogen). Medium was replaced 16 h after transfection. After 48 h, lentivirus-containing supernatants were collected, filtered through a 0.45 μm filter and added to bEnd.3 cells accompanied by Polybrene (10 μg ml^–1^). After 24 h, virus-containing medium was removed and replaced with fresh. Transduced cells were then sorted by FACS for dual expression of both P-selectin and GFP. To these sorted cells, mCherry-Cav1 (Plasmid no. 27705) was then introduced via transfection using Lipofectamine 3000. To facilitate selection of cells coexpressing both mCherry and GFP proteins, cells were sorted once more using FACS. Subsequently, double-positive cells were maintained in DMEM containing 10% FBS and 1% penicillin/streptomycin supplemented with 0.4 mg ml^–1^ G418 (Sigma), at 37 °C and under 5% CO_2_.

### shRNA-mediated gene knockdown

The pLKO.1 plasmids (Mission shRNA library, Sigma-Aldrich) were provided by the Gene Editing and Screening Core at Memorial Sloan Kettering Cancer Center (MSKCC). Lentivirus was prepared as previously described using pLKO.1 plasmids (Supplementary Table 5) designed for knockdown of Cav1 or CLTC, along with helper plasmids psPAX2 and pMD2.G. In the presence of Polybrene (10 μg ml^–1^), bEnd.3 cells were transduced with lentivirus for 24 h. Thereafter, puromycin (5 μg ml^–1^) was utilized to select for successfully transduced cells stably expressing shRNAs that mediate knockdown of CAV1 or CLTC, along with the puromycin-*N*-acetyltransferase resistance gene.

### Animals

All mice in this study were maintained under protocols approved by the Institutional Animal Care and Use Committee at Weill Cornell Medicine and MSKCC. SHH-MB mice (*Ptf1a*^*cre/+*^*;Ptch1*^*fl/fl*^) were generated by intercrossing *Ptf1a*^*cre/+*^ mice^31^ with *Ptch1*^*fl/fl*^ mice^32^ and maintained on a C57BL/6 background. *Cav1* null (JAX Stock no. 007083) and *Selp* null mice (JAX Stock no. 008432) were bred with SHH-MB mice to generate *Ptf1a*^*cre/+*^*;Ptch1*^*fl/fl*^*;Cav1*^*−/−*^ and *Ptf1a*^*cre/+*^*;Ptch1*^*fl/fl*^*;Selp*^*−/−*^ SHH-MB mice, respectively^33,34^. The genotype of each mouse was confirmed by PCR genotyping of a tail biopsy using primers for *Ptch1*, *Cre*, *Cav1 and Selp* (see Supplementary Table 3 for primer sequences). Both sexes were used for all studies. Animals were housed under a 12/12 h light/dark cycle and given access to food and water ad libitum. The numbers of tumours and/or mice analysed are provided in the main text and/or in the figure legends.

### Assessment of mouse SHH-MB BBB integrity

A bolus of 100 μl of a 10 mg ml^–1^ 70 kDa dextran-TMR solution (Life Technologies) was injected via the tail vein into tumour-bearing SHH-MB mice at advanced symptomatic stages, as previously described^6^. Brains were then removed 2 h later without perfusion, fixed overnight in 4% paraformaldehyde, embedded in optimal cutting temperature compound (O.C.T.) and then sections prepared at a thickness of 12 μm. Immunofluorescent staining of tissue sections was performed using antibodies against CD31 and P-selectin with appropriate secondary antibodies, counterstained with DAPI to visualize nuclei and then coverslipped using Fluoro-Gel mounting medium as described below. Detection of TMR-dextran in the context of P-selectin and CD31 immunostaining was imaged using a fluorescent microscope (Zeiss Axioobserver), and TIFF images postprocessed using Adobe Photoshop CS6.

### In vivo treatment studies

SHH-MB mice at advanced symptomatic stages (domed head, weight loss, ataxia) were treated with whole-body XRT using a RS2000 small animal irradiator (Rad Source) at the indicated doses (0, 0.25, 1 or 2 Gy) alone, with free vismodegib alone, with nanoparticle-encapsulated vismodegib (FiVis or DexVis) alone or in combinations as indicated in individual figure legends. When used in combination, drugs were administered via intraperitoneal injection 2 h following XRT administration and assayed at the indicated time points for immunoblot and RT–qPCR studies. Treatment for survival studies was given on alternate days for a total of eight doses starting on day 0, with all treatments in all experimental groups ending on day 16.

### Immunohistochemistry

Immunohistochemical staining of murine SHH-MB tissues was performed at the Molecular Cytology Core Facility of MSKCC. Brain tissues were harvested from SHH-MB mice and fixed in 4% paraformaldehyde overnight. Fixed tissues were embedded in O.C.T. and frozen sections prepared at a thickness of 12 μm. Heat antigen retrieval (95 °C for 20 min) was performed with citric acid buffer (pH 6.0), and sections were blocked for 30 min with 10% normal rabbit serum in PBS. Sections were incubated with primary antibodies (CD31, P-selectin, Cav1) overnight at 4 °C and secondary antibodies for 1 h at room temperature (see Supplementary Table 1 for antibody details). Slides were counterstained with Hoechst 33258 dye (Invitrogen) and coverslipped with Fluoro-Gel mounting medium (Electron Microscopy Sciences). Immunohistochemical detection of human SHH-MB tissue was performed at the Weill Cornell Medicine Center for Translational Pathology. Deidentified human SHH-MB tissue was molecularly characterized using genome-wide methylation classification approaches as previously described^35^. Tumour tissue was formalin fixed, paraffin embedded and prepared as 5 μm tissue sections. Immunophenotyping was performed on a Leica Bond III system using the modified protocol J. Sections were pretreated using heat-mediated antigen retrieval with sodium citrate buffer (pH 6.0, epitope retrieval solution 1) for 30 min. Sections were then incubated with P-selectin antibody (Lifespan Biosciences, no. LS-B3656) for 60 min at room temperature and detected using an alkaline phosphatase conjugated compact polymer system. Fast Red was used as the chromagen. Sections were then counterstained with haematoxylin and mounted with micromount. All images were taken with either a bright-field and fluorescence microscope (Zeiss Axio Observer) or digital Panoramic Slide Scanner (3D Histech, Budapest Hungary). TIFF images (with no compression) were postprocessed using Adobe Photoshop CS6.

### Immunoblotting and quantification

SHH-MB tissue was dissected from tumour-bearing mice following 2 Gy irradiation and homogenized through tissue sonication in tissue lysis buffer (50 mM Tris-HCl, 120 mM NaCl, 5 mM EDTA, 0.5% NP-40, 100 mM NaF, 2 mM Na_3_VO_4_, 10 mM Na_4_P_2_O_7_) supplemented with protease inhibitor (Sigma, no. P8849). Protein concentration was determined using the Pierce BCA protein assay kit (ThermoFisher), and proteins were separated by NuPAGE Novex 10% Bis-Tris precast gels (ThermoFisher) before electrophoretic transfer to polyvinylidene difluoride membranes. Membranes were incubated first with Odyssey Blocking Buffer (LI-COR Biosciences) and then with primary antibodies for P-selectin, p53 and GAPDH and the appropriate secondary antibodies, and analysed using near-infrared imaging with the LI-COR Odyssey CLx Imaging System. The antibodies used are described in Supplementary Table 2.

### RT–qPCR

Total messenger RNA was isolated from dissected mouse SHH-MB brain regions using TRI Reagent (Molecular Research Center). Reverse transcription was performed with the iScript cDNA synthesis kit (Bio-Rad), and qPCR using SsoAdvanced Universal SYBR(R) Green Supermix (Bio-Rad) according to the manufacturer’s instructions. Fold changes in expression were calculated using the ΔΔCT method. The *Gapdh* gene was used to normalize results. The primer sequences used are described in Supplementary Table 3.

### Nanoparticle fluorescence imaging and quantification

Nanoparticle localization in brains of SHH-MB mice was analysed ex vivo using a LI-COR Odyssey CLx Imaging System. Brains were scanned at a depth of 1 mm from the dorsal surface at 42 µm resolution in the 800 nm channel to detect fluorescence emission from the IRDye783 incorporated into FiVis nanoparticles. Images were quantified using Image Studio Software v.5.2.5 (LI-COR Biosciences). In brief, forebrain and cerebellar areas for quantification were demarcated and the relative signal per demarcated area was defined as the sum of the pixel density per demarcated area. The relative signal per demarcated area was normalized to pixel density signal from forebrain regions of untreated mice. For consistency, total cerebellar regions were demarcated, which included SHH-MB tumour regions, and compared with demarcated forebrain regions.

### Vismodegib bone toxicity studies

Juvenile C57BL/6 WT mice (P10) were administered either FiVis (10 or 20 mg kg^–1^) or free vismodegib (100 mg kg^–1^) twice daily for a total of four doses and compared with vehicle control mice at 6 weeks of age. The skeletal effects of each treatment were assessed by measurement of femur length using calipers. Microcomputed tomography analysis was conducted using a Scanco Medical microcomputed tomography 35 system at the Citigroup Biomedical Imaging Core as previously described^36^. Briefly, an isotropic voxel size of 7 µm was used to image the distal femur. Scans were conducted in 70% ethanol and used an X-ray potential of 55 kVp, an X-ray intensity of 0.145 mA and an integration time of 600 ms. Microcomputed tomography analysis was performed by an investigator blinded to the treatment of the animals under analysis. All endpoint microcomputed tomography analysis was carried out on 6-week-old mice.

### Nanoparticle transwell assay

Mouse brain endothelial cells (bEnd.3) were seeded on the upper surface of the membrane in polyester transwell inserts (0.4 μm pore size, 1 × 10^8^ cm^–2^ pore density, 8.4 mm diameter) at a density of 1 × 10^5^ cells per well. Media were changed every other day and cells cultured for 5–7 days until a confluent monolayer formed. Before initiation of transport studies, TEER across cell layers was measured until reaching a value of 30 Ω × cm^2^. Once bEnd.3 cell monolayers reached this threshold TEER, endothelial paracellular barrier function was evaluated by measuring the permeability of cells to 70 kDa TMR-dextran). The concentration of TMR-dextran was determined by measuring fluorescence (excitation at 555 nm and emission at 580 nm) using a TECAN plate reader. After confirming restriction to paracellular transport, transport studies with FiVis nanoparticles were carried out by the addition of 200 µl of FiVis nanoparticles (20 µg ml^–1^) to the upper chamber of the insert. At various time points thereafter, the entire basal well volume was removed and assayed for nanoparticle concentration by measurement of fluorescence (excitation at 790 nm and emission at 815 nm), and for nanoparticle size using DLS.

### Mass spectrometry for quantification of vismodegib in brain tissue

Analysis of vismodegib concentrations in brain tissue was performed by Integrated Analytical Solutions. As with efficacy studies, mice were irradiated with 0.25 Gy XRT. Two hours later, mice were injected intraperitoneally with nanoparticles containing 10 mg kg^–1^ vismodegib. After 4 h, mice were sacrificed and brain tissue was sectioned into two regions: forebrain (normal tissue) and cerebellum (tumour tissue). The tissue of each region was weighed and snap-frozen before LC–MS analysis by Integrated Analytical Solutions. A standard curve was generated by the addition of known amounts of vismodegib to homogenized brain tissue from nontreated mice. The concentration of vismodegib in forebrain and cerebellar tumour tissue samples was then calculated to determine the mass of vismodegib (ng) per gram of tissue.

### Statistics and reproducibility

All data are shown as either mean ± s.d. or mean ± s.e.m., unless otherwise indicated. For comparison between two groups, unpaired, two-tailed Student’s *t-*tests were used. Analysis of variance (ANOVA), followed by a post hoc test for multiple comparisons (Dunnett’s), was used for comparison of groups of three or more. For Kaplan–Meier survival analysis, the log-rank (Mantel–Cox) test was used. GraphPad Prism v.9.1.0 software was used for statistical analysis. Analysis of fluorescence in histology samples was processed in QuPath v.0.1.3 (Queen’s University, Belfast, UK)^37^. *P* < 0.05 was considered statistically significant, and additional indicators of statistical significance are provided accordingly in the text or in individual figure legends. Sample sizes were chosen following consultation with our biostatistics collaborator, and based on previous literature in SHH-MB tumour biology and nanomedicine. All in vivo experiments were performed at a minimum of *n* = 3 to validate the results for each treatment group. Assignment of sick mice to a treatment group was random. Because in vivo experiments addressed sex as a biological variable, both male and female mice were included in all mouse studies. For in vitro studies, we performed experiments with a minimum of *n* = 3 biologically independent groups derived from distinct wells per condition tested. We found this sample size sufficient to control for any technical variations, and extensive experience has shown it to be sufficient to determine reproducible results from cultured cells. All experiments were reproduced to reliably support conclusions stated in the manuscript and were performed with explicit considerations of caveats of experimental models, including appropriate control groups and variables to ensure robustness of results.

### Ethics statement

All mice in this study were maintained under protocols approved by the Institutional Animal Care and Use Committee at Weill Cornell Medicine and Memorial Sloan Kettering Cancer Center. Maximal tumour symptomatic burden, including weight loss and ataxia as defined in these approved protocols, was assessed daily through all treatment studies. Maximal symptomatic tumour burden was not exceeded and mice were euthanized humanely as per the approved institutional protocols.

### Reporting summary

Further information on research design is available in the Nature Portfolio Reporting Summary linked to this article.

## Online content

Any methods, additional references, Nature Portfolio reporting summaries, source data, extended data, supplementary information, acknowledgements, peer review information; details of author contributions and competing interests; and statements of data and code availability are available at 10.1038/s41563-023-01481-9.

## Supplementary information

Supplementary Information Supplementary Figs. 1–19 and Tables 1–5.

Reporting Summary

Supplementary Video 1 Time lapse acquired by confocal microscopy showing live cell imaging of fluorescent P-selectin, Cav1 and nanoparticles.

Supplementary Data 1 Statistical Source Data for Supplementary Fig. 1.

Supplementary Data 2 Statistical Source Data for Supplementary Fig. 2a.

Supplementary Data 2 Unprocessed immunoblot image for Supplementary Fig. 2b.

Supplementary Data 3 Statistical Source Data for Supplementary Fig. 3.

Supplementary Data 4 Statistical Source Data for Supplementary Fig. 4.

Supplementary Data 5 Statistical Source Data for Supplementary Fig. 5.

Supplementary Data 6 Statistical Source Data for Supplementary Fig. 6.

Supplementary Data 8 Statistical Source Data for Supplementary Fig. 8.

Supplementary Data 9 Statistical Source Data for Supplementary Fig. 9.

Supplementary Data 10 Statistical Source Data for Supplementary Fig. 10.

Supplementary Data 15 Statistical Source Data for Supplementary Fig. 15.

Supplementary Data 17 Statistical Source Data for Supplementary Fig. 17.

Supplementary Data 18 Statistical Source Data for Supplementary Fig. 18.

Supplementary Data 19 Statistical Source Data for Supplementary Fig. 19.

## Data Availability

All referenced data in this manuscript are available either within the main article file or the Supplementary Materials provided. The authors have also provided source data for all figures presented in the main manuscript, Extended Data and Supplementary Information. Upon reasonable request, additional data related to this study can be made available from the corresponding authors. Source data are provided with this paper.

## Source data

Source Data Fig. 1 Statistical Source Data for Fig. 1.
Source Data Fig. 2 Statistical Source Data for Fig. 2.
Source Data Fig. 3 Statistical Source Data for Fig. 3.
Source Data Fig. 4 Statistical Source Data for Fig. 4.
Source Data Fig. 5 Statistical Source Data for Fig. 5.
Source Data Extended Data Fig. 2 Statistical Source Data for Extended Data Fig. 2.
Source Data Extended Data Fig. 3 Unprocessed imunoblot images.
Source Data Extended Data Fig. 4 Statistical Source Data for Extended Data Fig. 4.
Source Data Extended Data Fig. 5 Statistical Source Data for Extended Data Fig. 5.
Source Data Extended Data Fig. 6 Statistical Source Data for Extended Data Fig. 6.
Source Data Extended Data Fig. 7 Statistical Source Data for Extended Data Fig. 7.
Source Data Extended Data Fig. 8 Statistical Source Data for Extended Data Fig. 8.
Source Data Extended Data Fig. 9 Statistical Source Data for Extended Data Fig. 9.
Source Data Extended Data Fig. 10 Statistical Source Data for Extended Data Fig. 10.
