# Supplementary material for: P-selectin-targeted nanocarriers induce active crossing of the blood–brain barrier via caveolin-1-dependent transcytosis
Source: Nat Mater. 2023 Mar 2;22(3):391–9. doi: 10.1038/s41563-023-01481-9 (PMC9981459; doi:10.1038/s41563-023-01481-9)
Supplement: Supplementary file 1 — Supplementary Figs. 1–19 and Tables 1–5. [file 41563_2023_1481_MOESM1_ESM.pdf]

nature materials

Article

https://doi.org/10.1038/s41563-023-01481-9

P-selectin-targeted nanocarriers induce active crossing of the blood–brain barrier via caveolin-1-dependent transcytosis

In the format provided by the authors and unedited

Table of Contents for Supplementary Information

	Page(s)
**Supplementary Figures** (1-19)	2-20
**Supplementary Tables** (1-5)	21-25

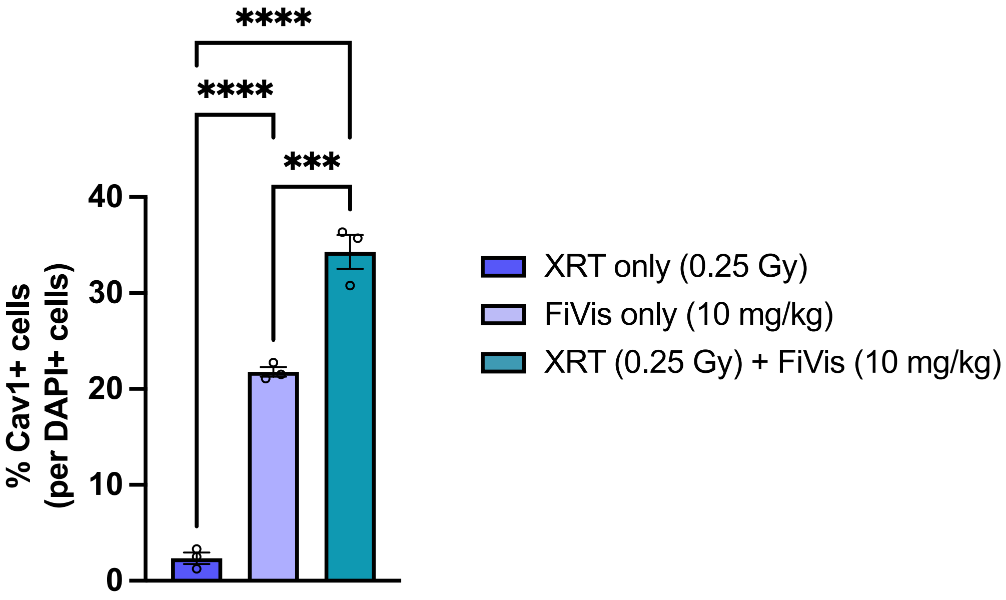

Supplementary Fig. 1: Quantification of Cav1 expression in SHH-MB mouse tissue. (**a**) Percentage of cells expressing Cav1, normalized to DAPI signal. Data in are means ± SEM. ***P < 0.001, ****P < 0.0001 (one-way ANOVA). Following treatment with XRT only, FiVis only, or a combination of XRT + FiVis, immunofluorescence histology and analysis was performed on distinct brain tissue slices (n=3). For each treatment group, Cav1 expression was quantified within ROIs that encompass tumor regions.

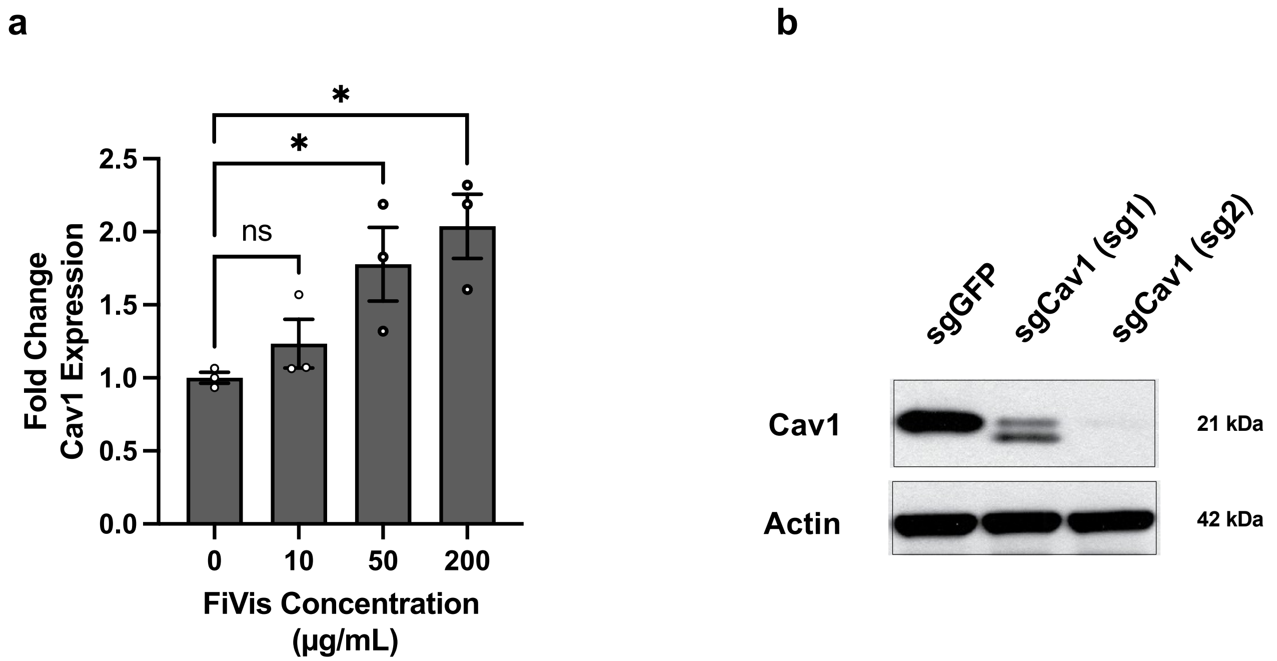

Supplementary Fig. 2: **Modulation of caveolin-1 expression in bEnd.3 cells.** (**a**) Cav1 expression in bEnd.3 cells measured upon incubation with FiVis nanoparticles, by qPCR. Data are means ± SEM. *n* = 3 biologically independent samples; **P* < 0.05 (two-tailed t test); ns, not significant. (**b**) Western blot for Cav1 expression following CRISPR-Cas9–mediated knockout of caveolin-1 in bEnd.3 cells. Data are means ± SEM, **P* <0.05 (unpaired t test). ns, not significant.

a

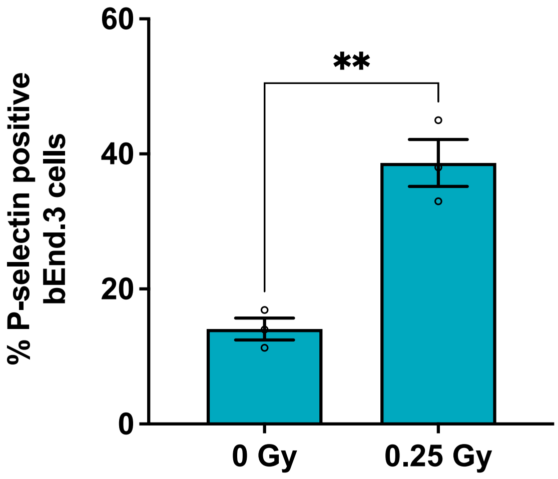

b

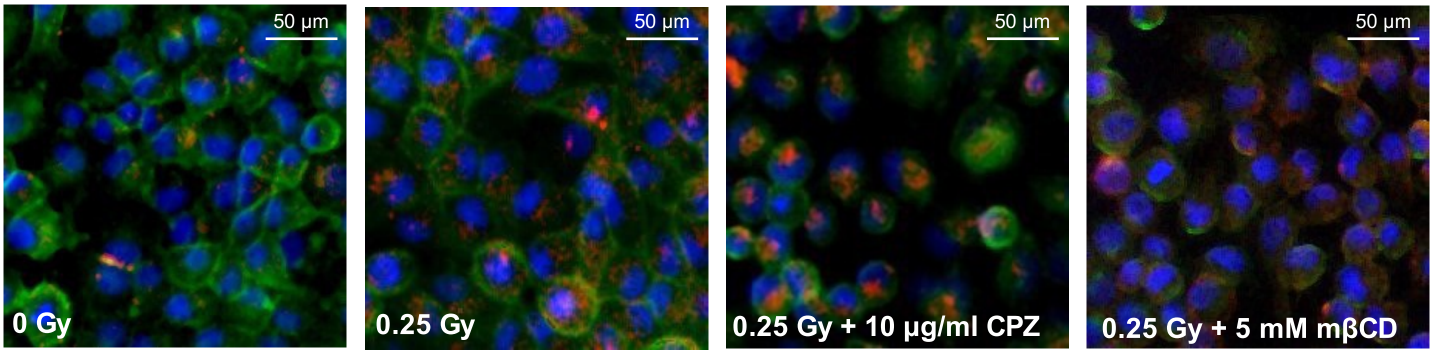

Supplementary Fig. 3: **bEnd.3 endothelial cell P-selectin expression and FiVis nanoparticle uptake after XRT.** (**a**) Murine brain endothelial cells (bEnd.3) assayed by flow cytometry for surface P-selectin expression following 0.25 Gy irradiation. The percentage of live cells expressing P-selectin is shown for non-irradiated (0 Gy) and irradiated (0.25 Gy) cells. Data are means ± SEM. $n = 3$ biologically independent samples; $^{**}P < 0.01$ (P=0.0030, two-tailed t test). (**b**) Fluorescence images of bEnd.3 cells incubated with FiVis nanoparticles. Non-irradiated control cells (left) were cultured using standard media. Irradiated groups received 0.25 Gy XRT and were cultured using standard media or media containing endocytosis inhibitors. CPZ = Chlorpromazine (inhibitor of clathrin-mediated endocytosis); CD = methyl-β-cyclodextrin (inhibitor of caveolin-dependent endocytosis). Color Legend: Red = IR-783/820 dye in FiVis nanoparticles; Green = CellMask Green plasma membrane stain; Blue = DAPI nuclear stain.

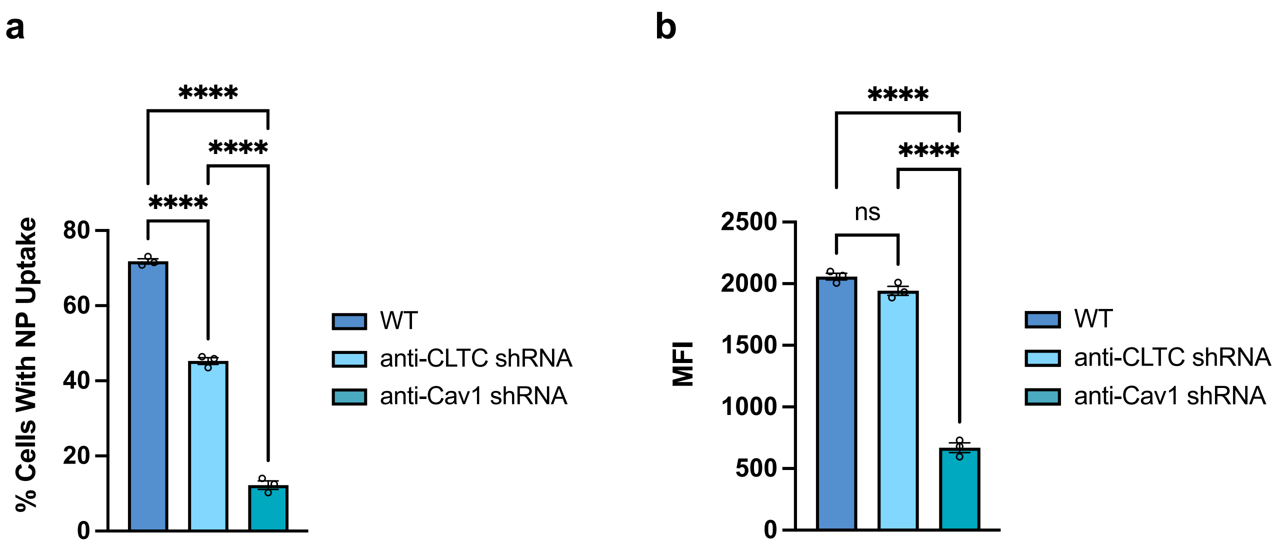

Supplementary Fig. 4: FiVis nanoparticle uptake in bEnd.3 cells after shRNA-mediated knockdown of clathrin heavy chain or caveolin-1 in bEnd.3 cells. (**a**) Percent uptake and (**b**) mean fluorescence intensity (MFI) of FiVis nanoparticles in WT bEnd.3 or shRNA expressing bEnd.3 cells. Data in (**a, b**) are means ± SEM. $n = 3$ biologically independent samples; ****$P < 0.001$ (one-way ANOVA); ns, not significant.

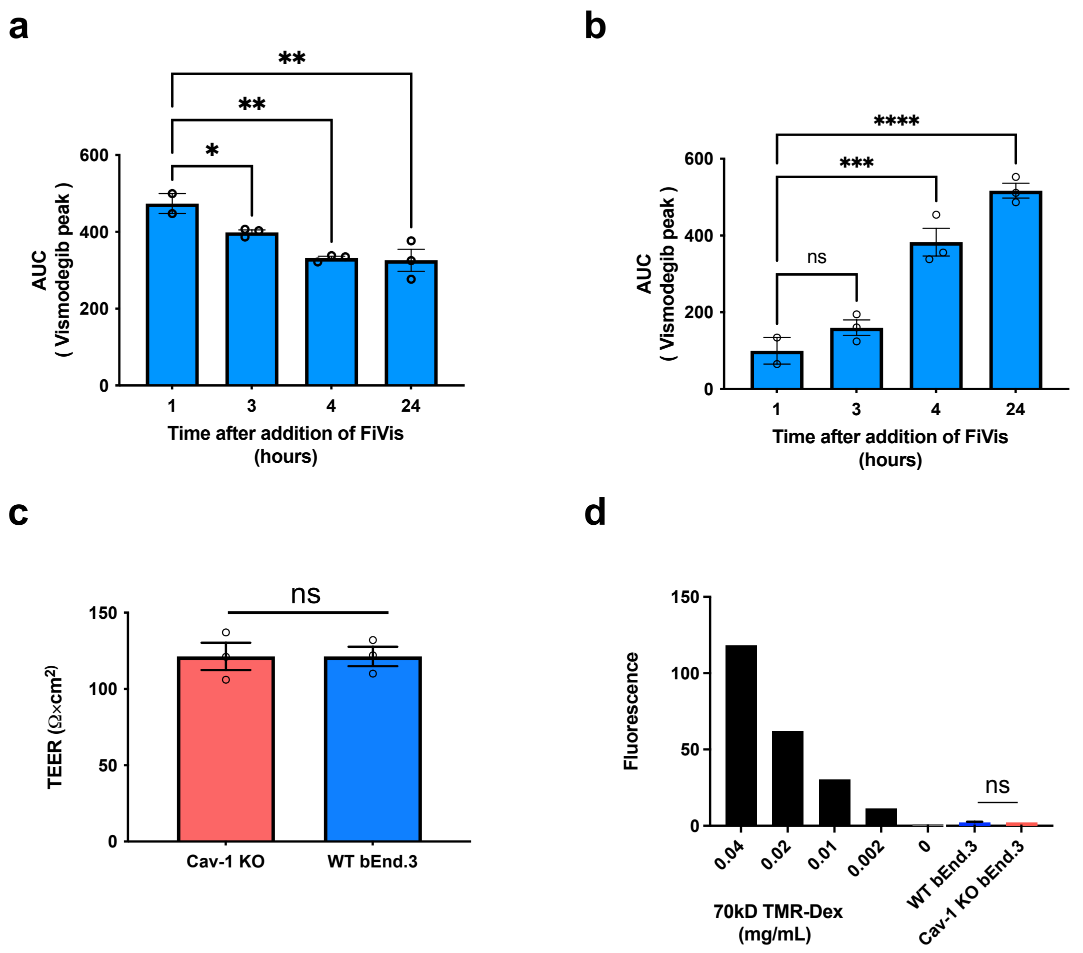

Supplementary Fig. 5: **Transwell assay for FiVis nanoparticle passage across murine brain endothelial cells.** Quantification of FiVis passage across transwell membrane inserts. HPLC quantification of vismodegib in the (**a**) top chamber or (**b**) bottom chamber of the well after introduction to the top chamber (**c**) Trans-endothelial electrical resistance (TEER) measurements of wildtype (WT) and caveolin-1 KO (Cav1 KO) bEnd.3 cells prior to incubation with FiVis nanoparticles. Data are means ± SEM. *n* = 3 biologically independent samples; ns, not significant (two-tailed t test). (**d**) Fluorescence quantification of 70 kDa TMR-Dextran passage into bottom chamber without cells (first 5 bars) and across a monolayer of WT or Cav1 KO bEnd.3 cells (last 2 bars, using a 0.5 mg/mL preparation). Data are means ± SEM. *n* = 3 biologically independent samples; ns, not significant (two-tailed t test)

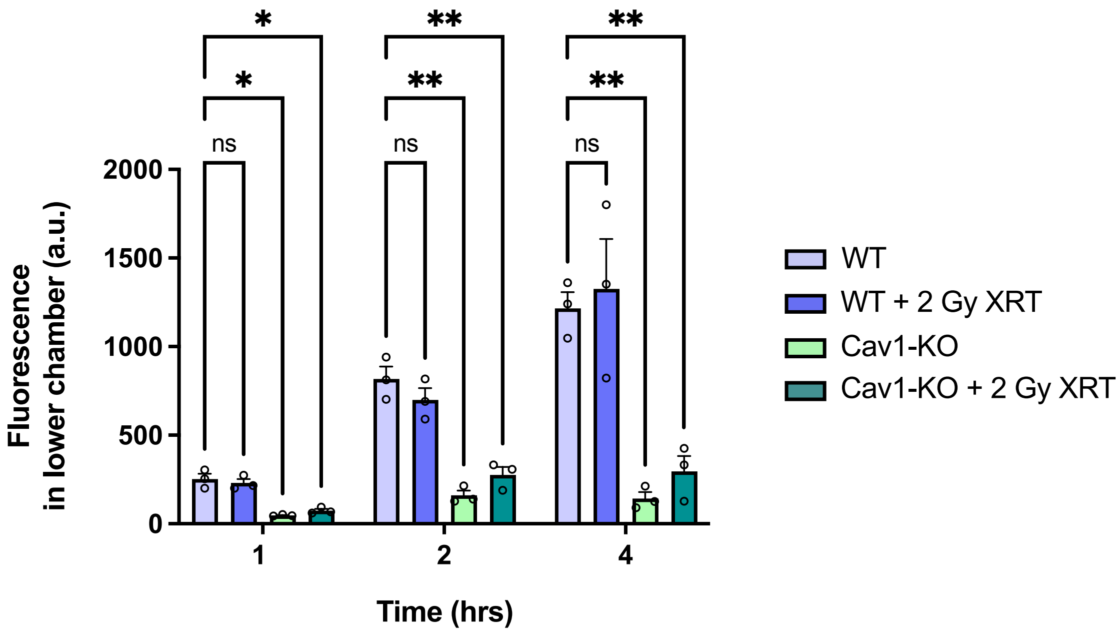

Supplementary Fig. 6: **Transwell assay of FiVis transport in irradiated bEnd.3 cells.** Wildtype bEnd.3 cells and Cav1-KO bEnd.3 cells pretreated with 2 Gy XRT or kept under normal conditions (non-irradiated) prior to incubation with FiVis nanoparticles. Fluorescence corresponding to FiVis nanoparticles (820 nm) measured in the lower chamber of the transwell membrane insert on which cells were cultured as monolayers. Measurements were taken at 1, 2, and 4 hours after nanoparticles were added to the apical side of the chamber. Data are means ± SEM. $n = 3$ biologically independent samples; $*P < 0.05$, $**P < 0.01$, (two-way ANOVA); ns, not significant.

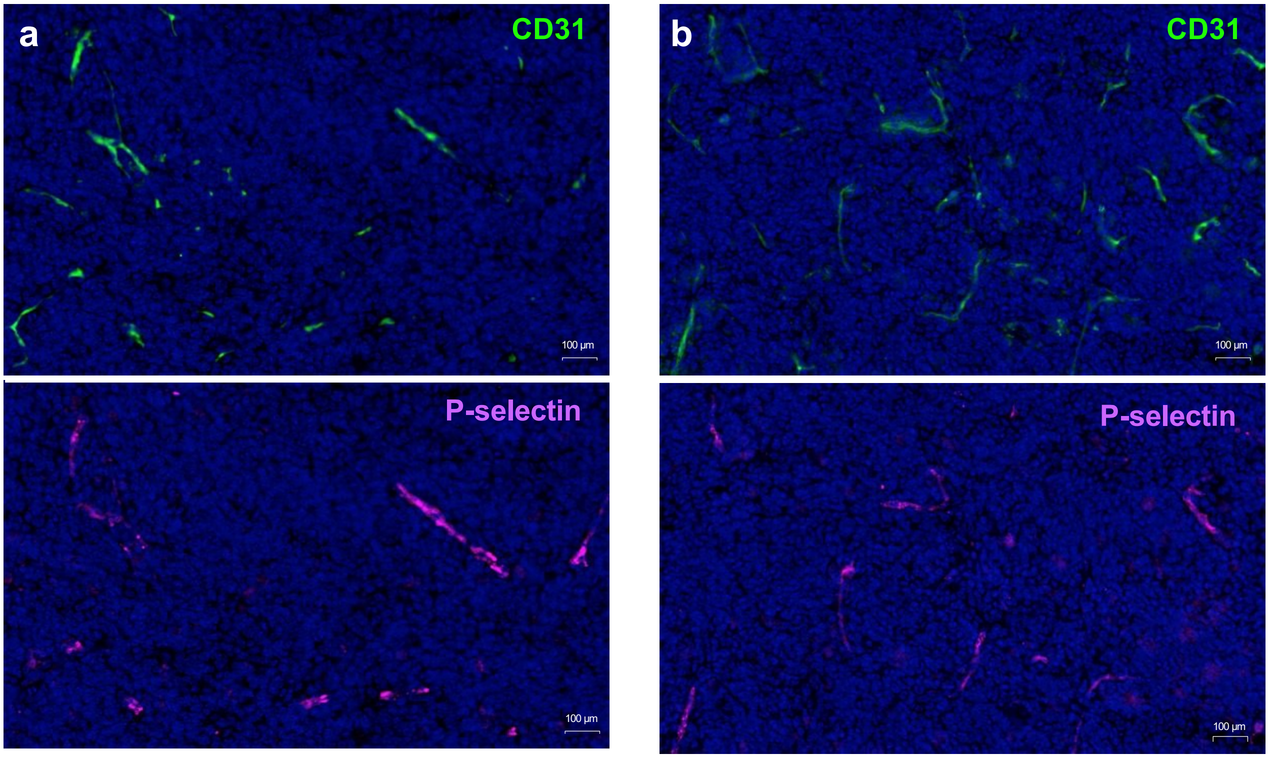

Mouse SHH MB *Ptf1a*$^{cre/+}$; *Ptch1*$^{fl/fl}$; *Cav1*$^{-/-}$

Mouse SHH MB *Ptf1a*$^{cre/+}$; *Ptch1*$^{fl/fl}$

Supplementary Fig. 7: **P-selectin expression in *Cav1*$^{-/-}$ SHH-MB mice.** Immunofluorescence stains of P-selectin in (**a**) homozygous Cav1 null SHH MB and (**b**) wild-type Cav1 tumor tissue. Green = CD31, endothelial cells, Violet = P-selectin, and blue = DAPI, nuclei.

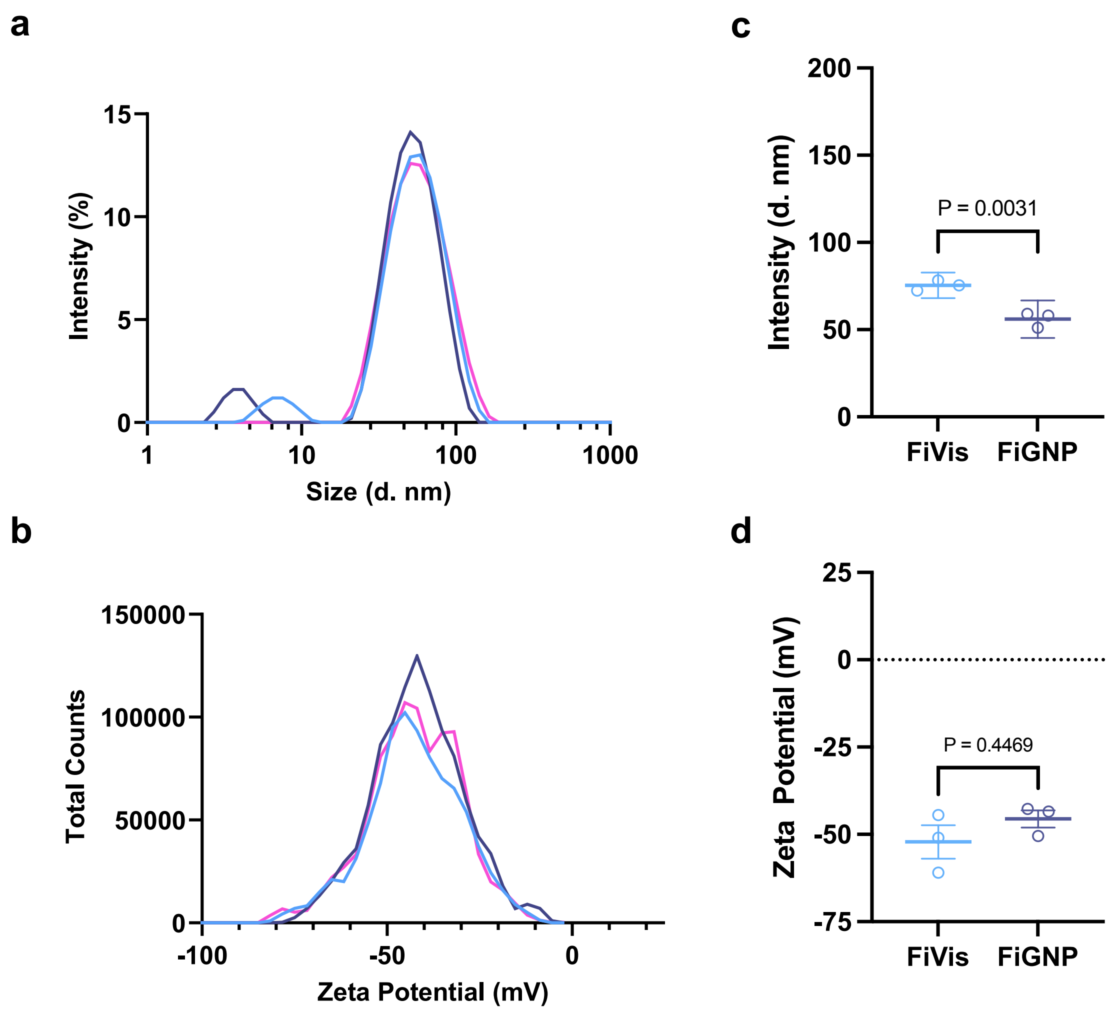

Supplementary Fig. 8: Characterization of fucoidan-coated gold nanoparticles (FiGNP). (**a**) Size (DLS) and (**b**) surface charge (zeta potential) measurements of FiGNPs. Comparison of (**c**) DLS and (**d**) zeta potential of FiVis and FiGNP nanoparticles. Data in (**c, d**) are means ± SEM. n = 3 independent experimental samples; ns, not significant (two-tailed t test).

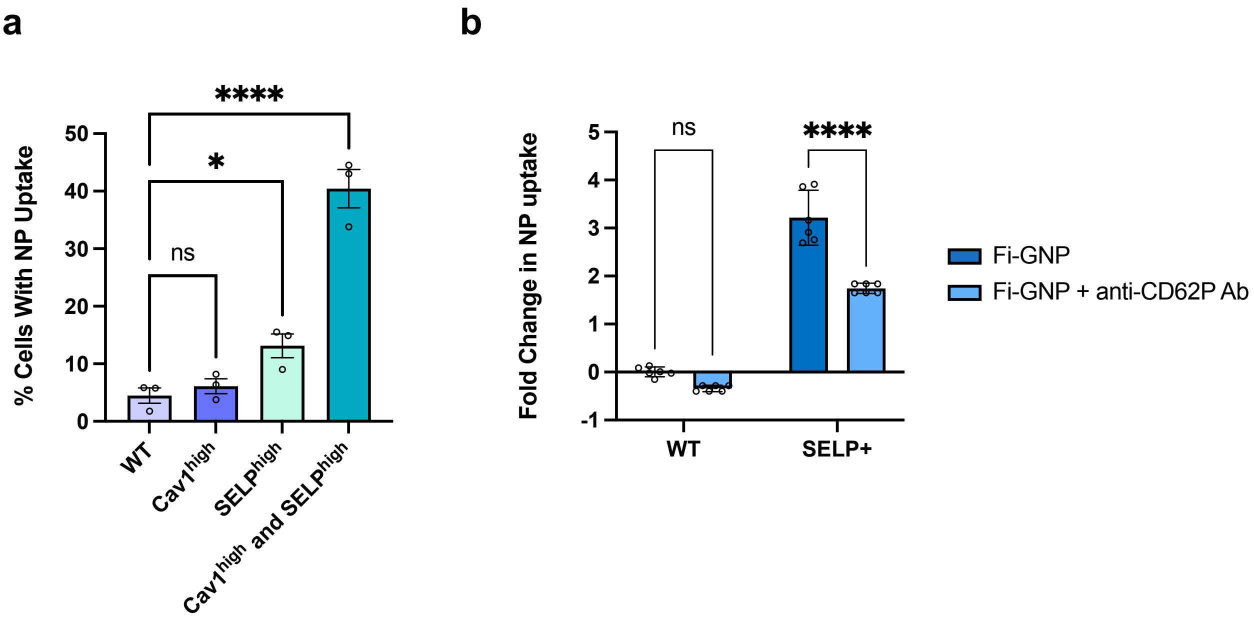

Supplementary Fig. 9: Fucoidan-functionalized gold nanoparticle (FiGNP) uptake in P-selectin-expressing bEnd.3 cells. (**a**) Uptake of FiGNPs assessed by flow cytometry in bEnd.3 cells modified to overexpress either caveolin-1, P-selectin, or both. Data are means ± SEM. $n = 3$ biologically independent samples; *$P < 0.05$, **$P < 0.01$, ****$P < 0.0001$ (one-way ANOVA); ns, not significant. **(b)** FiGNPs incubated in the presence or absence of anti-P-selectin (CD62P) antibody. Uptake of FiGNPs assessed in both WT and P-selectin overexpressing ($SELP^{high}$) bEnd.3 cells. Fold change in uptake was normalized to FiGNP uptake in WT cells without antibody. Data are means ± SEM. $n = 3$ biologically independent samples; ****$P < 0.0001$ (two-way ANOVA); ns, not significant.

a

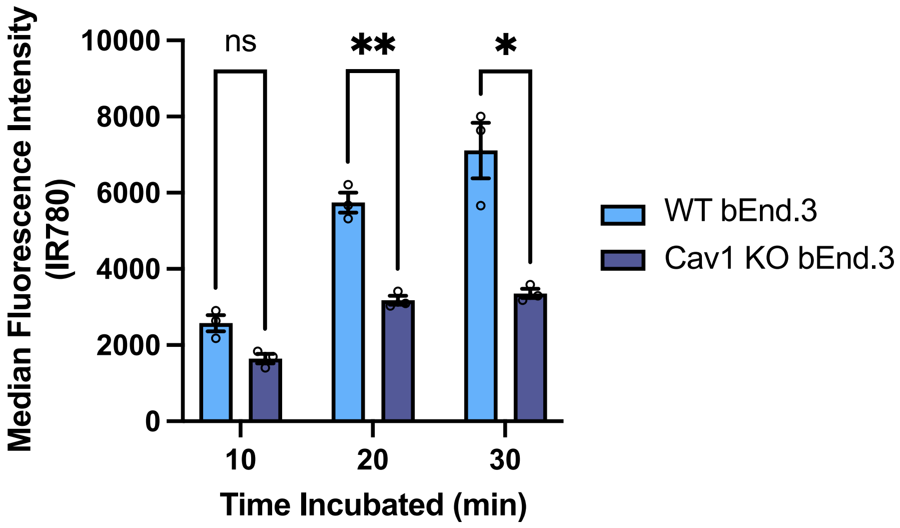

b

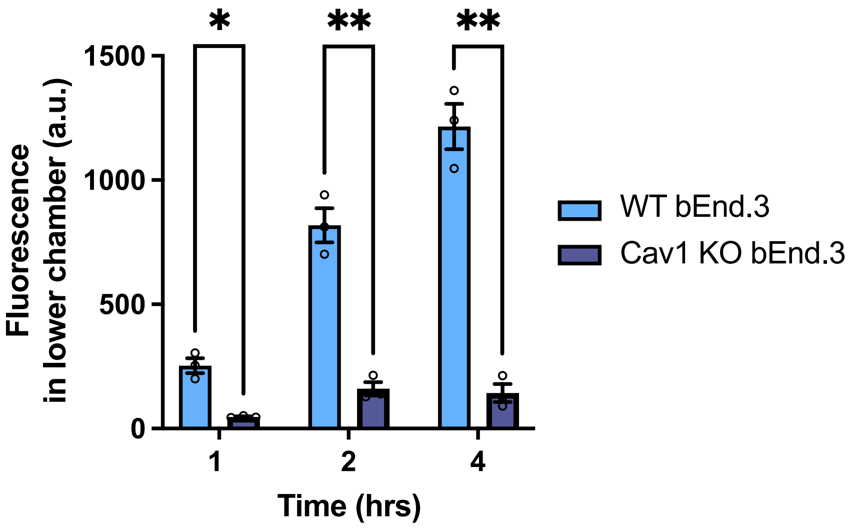

Supplementary Fig. 10: Transwell assay of FiGNPs and dependence on caveolin-1 expression. (a) Time course of FiGNP uptake into WT or Cav1-KO bEnd.3 cells, measured via flow cytometry. Median fluorescence intensity of IR780 is shown. (**b)** Quantification of FiGNP transport in a transwell plate seeded with a monolayer of WT or Cav1-KO bEnd.3 cells, measured at indicated times. Data shows IR780 fluorescence intensity measured in the lower chamber of the wells. Data in (**a, b**) are means ± SEM. $n = 3$ biologically independent samples; *$P < 0.05$, **$P < 0.01$(one-way ANOVA); ns, not significant.

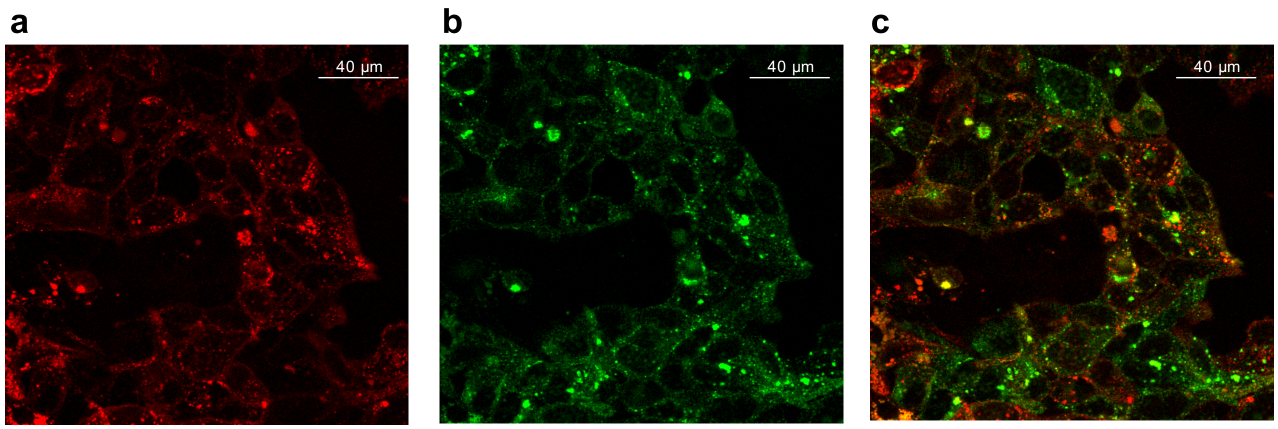

Supplementary Fig. 11: **Confocal microscopy of bEnd.3 cells engineered to express fluorescent caveolin-1 and P-selectin fusion proteins.** Murine bEnd.3 endothelial cells modified to express fluorescently-tagged caveolin-1 (mCherry-Cav1) and P-selectin (GFP-SELP) imaged by confocal microscopy to visualize (**a**) mCherry (red), (**b**) GFP (green), (**c**) and a merged image of the two channels.

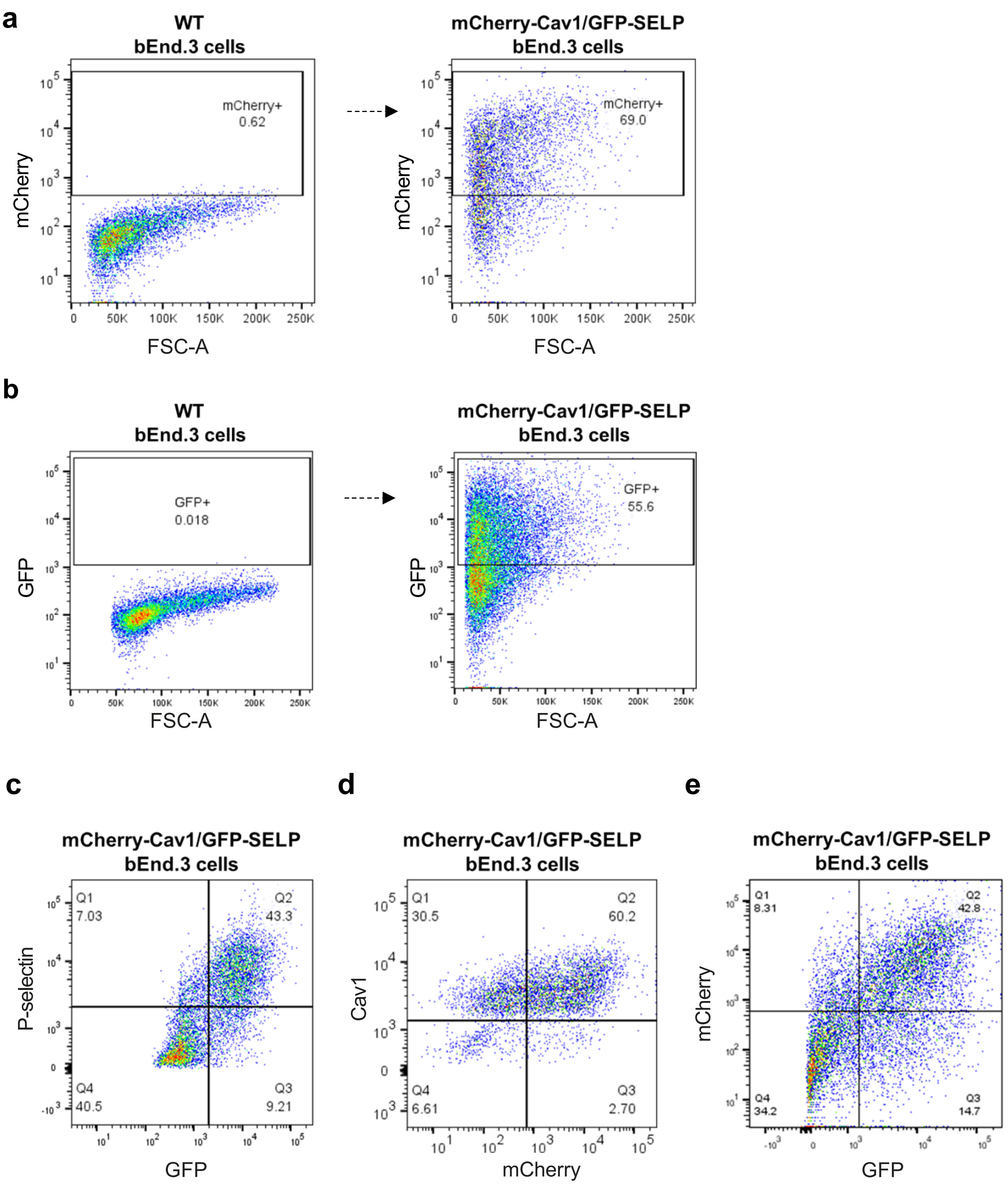

Supplementary Fig. 12: Validation of P-selectin and Caveolin-1 co-expression in modified bEnd.3 cells. WT and mCherry-Cav1/GFP-SELP modified bEnd.3 cells analyzed by flow cytometry for emission of (**a**) mCherry (Cav1) and (**b**) GFP (SELP). Flow cytometry analysis of mCherry-Cav1/GFP-SELP modified bEnd.3 cells using concomitant emission of both fluorescent protein tags and fluorescent antibodies. (**c**) P-selectin expression quantified using GFP and an anti-P-selectin antibody tagged using the fluorophore PE-Cy7. (**d**) Caveolin-1 expression measured using the mCherry tag and an anti-caveolin-1 antibody bound to the fluorophore BV-421. (**e**) mCherry-caveolin-1 and GFP-P-selectin emission.

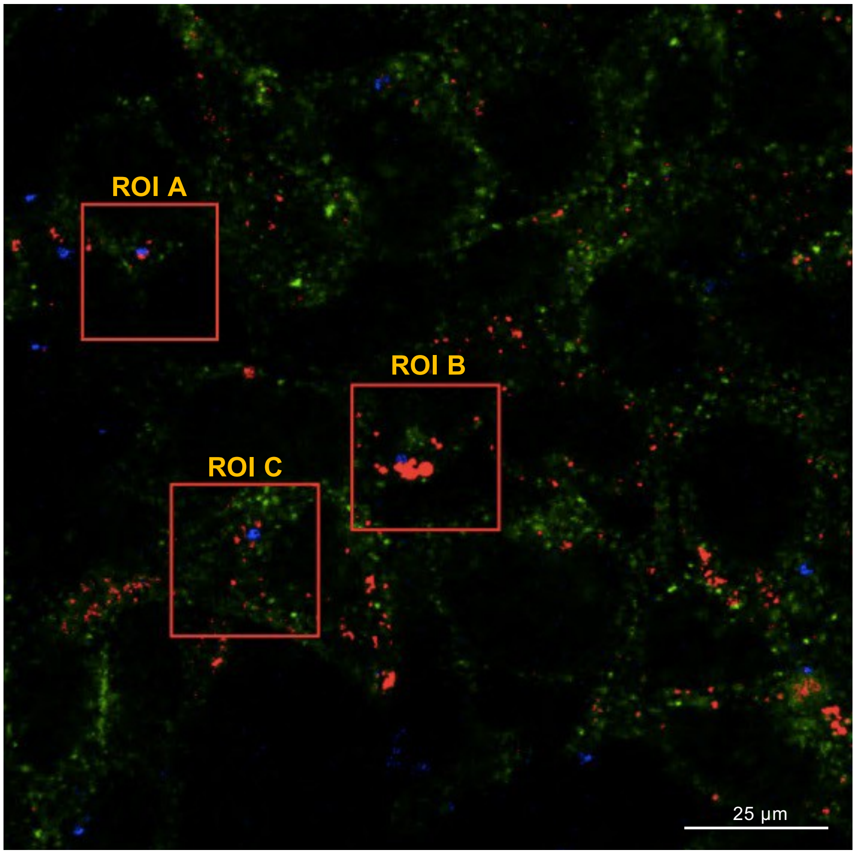

Supplementary Fig. 13: Confocal live cell imaging of FiGNPs in mCherry-Cav1/GFP-SELP bEnd.3 cells. Red = mCherry-tagged caveolin-1; Green = GFP-tagged P-selectin; Blue = FiGNPs. The regions of interest (ROI) highlight regions showing apparent caveolin-1 interaction with FiGNP nanoparticles.

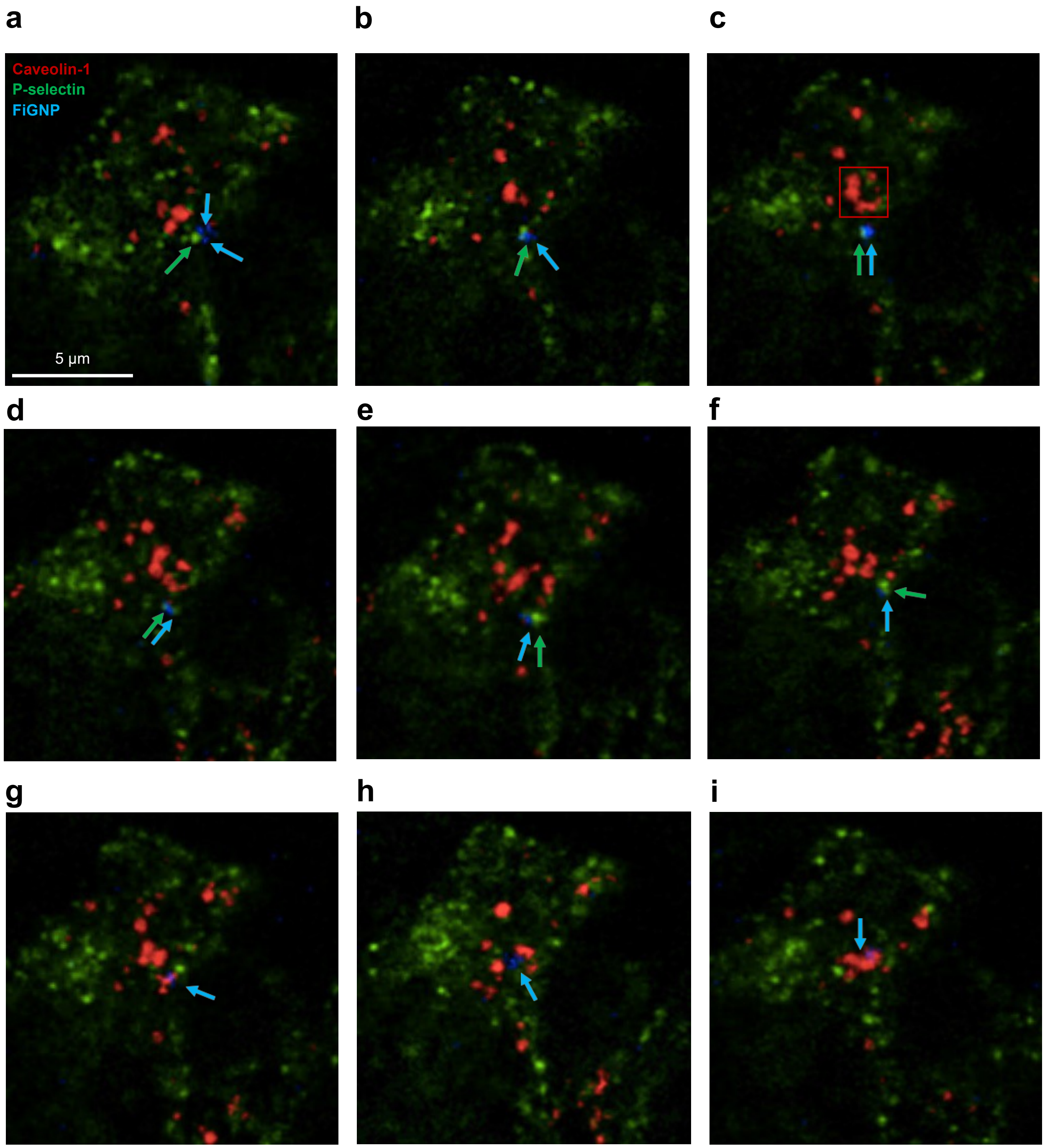

Supplementary Fig. 14: Fucoidan-coated nanoparticle engagement with P-selectin and caveolin-1 on the cell surface. Still images taken at 12 second intervals from Supplementary Video 1 (**a**) FiGNP particle (indicated by blue arrows) association with P-selectin (puncta indicated by green arrow). (**b**) Apparent FiGNP interaction directly with P-selectin (indicated by co-localizing fluorescent signals). (**c**-**f**) Prolonged association of FiGNP with P-selectin. (**g**) Association of FiGNP with caveolin-1. (**h**) FiGNP flanked by caveolin-1. (**i**) Caveolin-1 clustered around a FiGNP. Red = mCherry-tagged caveolin-1; Green = GFP-tagged P-selectin; Blue = FiGNPs. Scale bar = 5 μm.

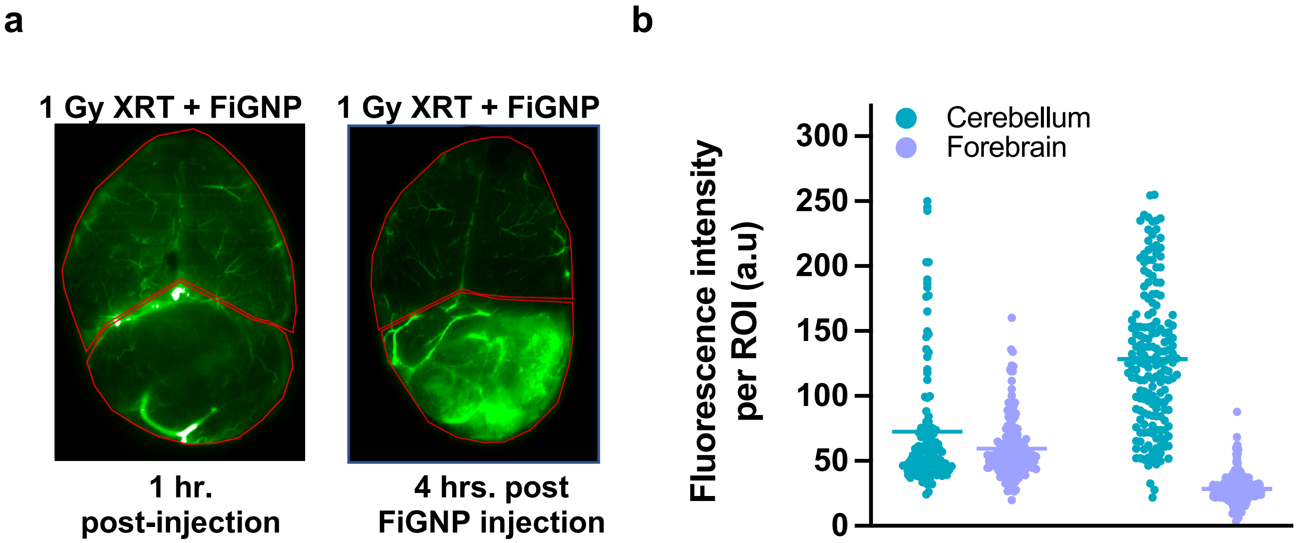

Supplementary Fig. 15: FiGNP imaging in brains of SHH-MB mice. (**a**) Near-IR images of brains harvested 1 h (left) or 4 h (right) after FiGNP administration (70 µg/mL, 1.5×10^{10} NP/mL). Mice were injected two hours after irradiation with 1 Gy XRT, SHH-MB mice were injected intraperitoneally with 200 µl of FiGNP suspension at a concentration of 70 µg/mL. (**b**) Quantification of near-IR fluorescence in cerebellum or forebrain from the images in (**a**)**.** Data points represent mean fluorescence intensity per randomly generated regions of interest (n > 100) within the delineated brain areas (red) for each treatment group.

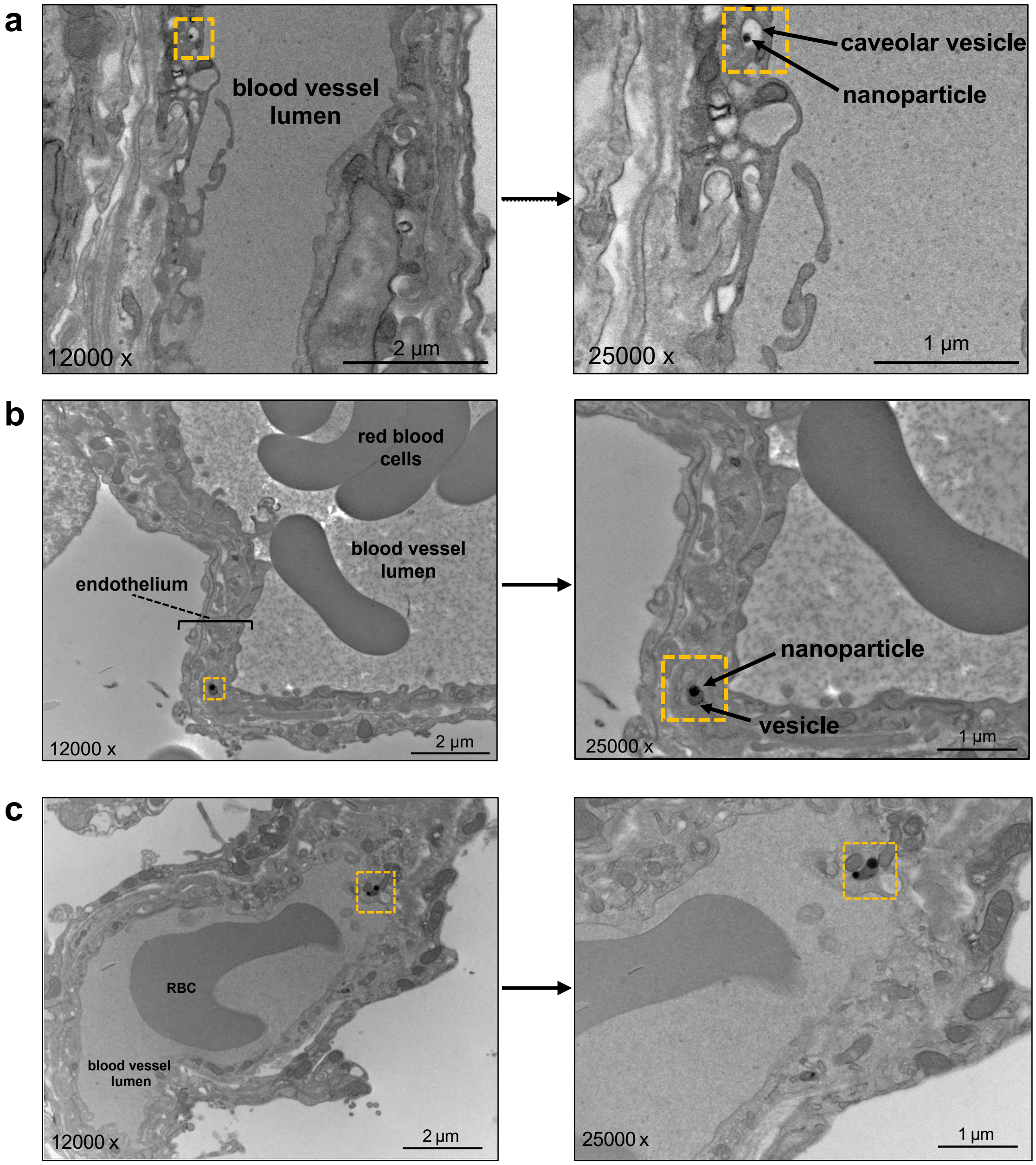

Supplementary Fig. 16: **TEM imaging of fucoidan-functionalized gold nanoparticles in SHH-MB mouse brains**. (**a-c**) TEM images of cerebellar tumor tissue sections from SHH-MB mouse brains. Tissues were resected 1 h after FiGNP injection (70 µg/ml; 1.5×10^{10} NP/mL), and 3 h after administration of 1 Gy XRT. Images taken at 12,000 x magnification (left) are shown with corresponding images taken at 25,000 x magnification (right).

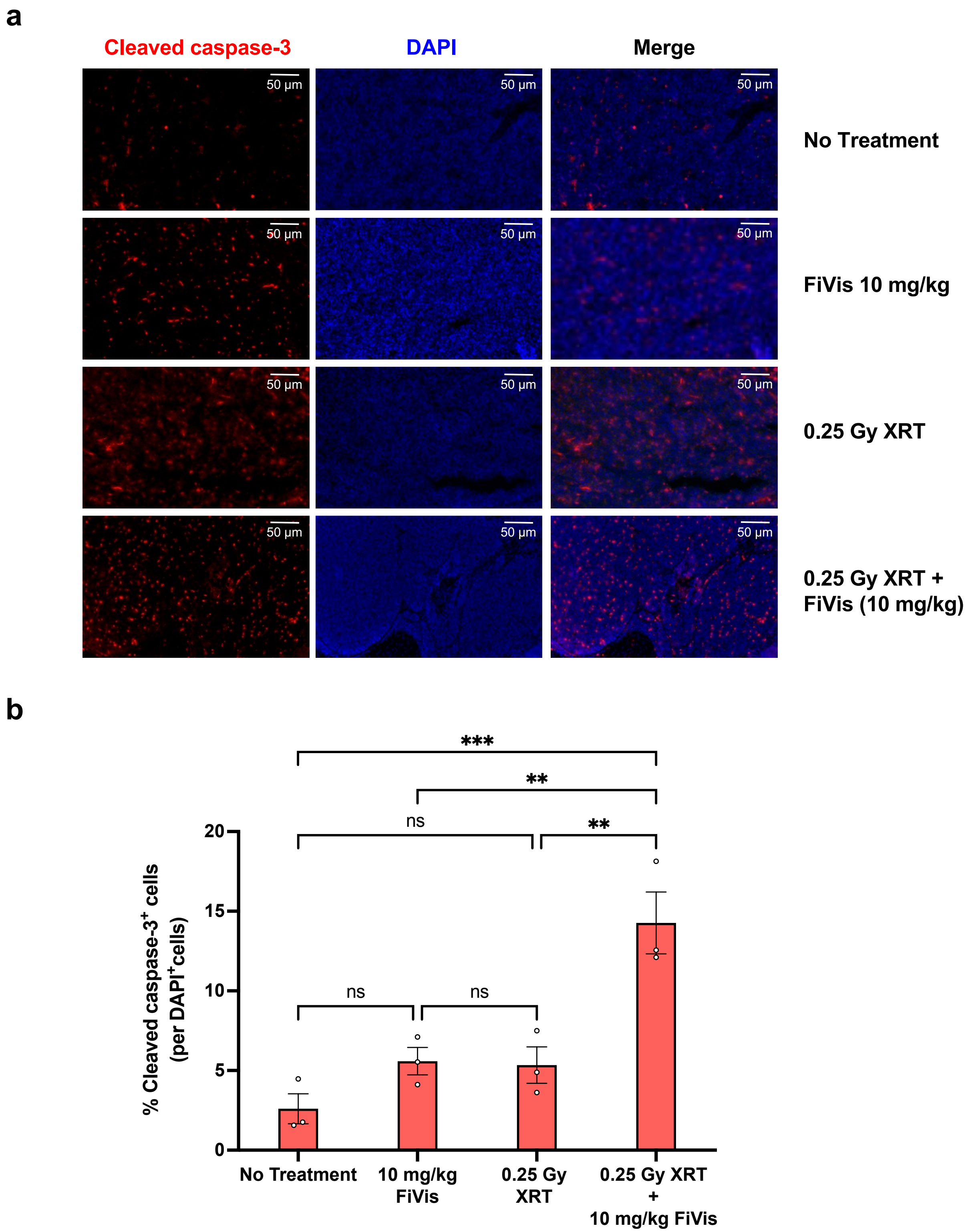

Supplementary Fig. 17 Cleaved caspase-3 expression in SHH-MB tumors following treatment with ionizing radiation and FiVis nanoparticles. Representative images (**a**) and quantification (**b**) of immunofluorescent staining for cleaved caspase-3 (red) and DAPI (blue) in advanced SHH-MB mouse tumors of nontreated mice or following treatment with 10 mg/kg FiVis, 0.25 Gy XRT, or a combination 0.25 Gy XRT and 10 mg/kg FiVis. Data in (**b**) are means ± SEM. $*P < 0.05$, $**P < 0.01$, $***P < 0.001$ (one-way ANOVA); ns, not significant. For n=2 mice per treatment group, 3 random regions of interest within SHH-MB tumor areas were used to quantify the percentage of cleaved caspase-3 positive cells per total number of DAPI positive nuclei.

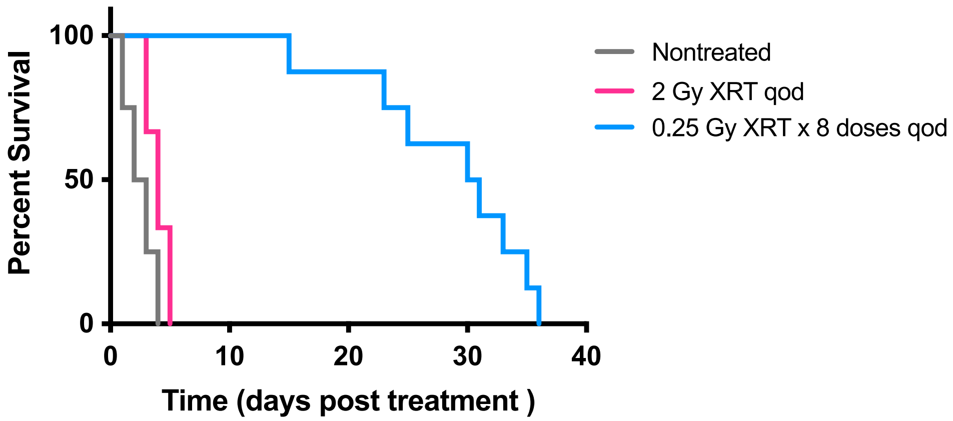

Supplementary Fig. 18: Survival of SHH-MB mice treated with 0.25 Gy XRT or 2 Gy XRT. Mice with SHH-MB were treated with either 2 Gy or 0.25 Gy of ionizing radiation and dosed every other day (q. o.d.) for up to 8 doses.

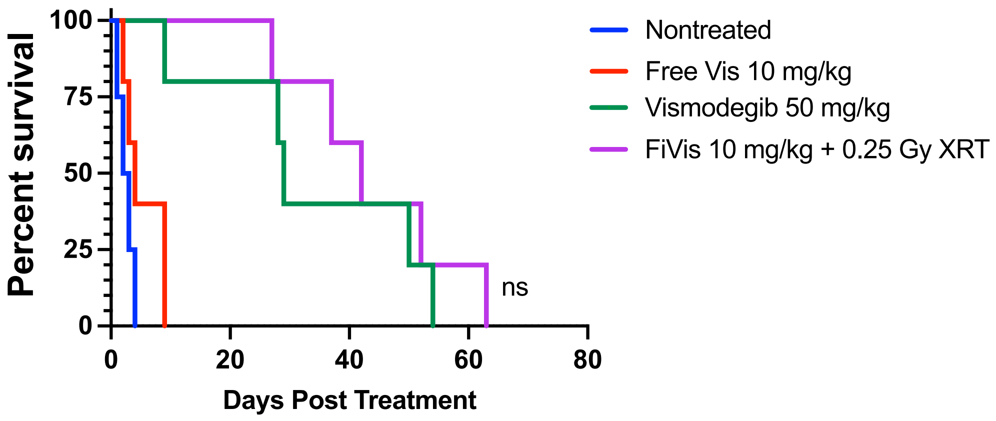

Supplementary Fig. 19: **Survival of SHH-MB mice treated with free vismodegib and FiVis nanoparticles.** Kaplan-Meier survival analysis of advanced stage SHH-MB mice treated with either 10 mg/kg free vismodegib (red), 50 mg/kg free vismodegib (green), or low dose ionizing radiation combined with FiVis nanoparticle (violet) at indicated doses given every other day for up to 8 doses. Survival of untreated advanced stage SHH-MB mice is shown in blue. Statistical comparisons between survival curves were performed using a log-rank (Mantel-Cox) test. *P* = ns for the comparison of groups treated vismodegib 50 mg/kg (green) and 0.25 Gy XRT + FiVis 10 mg/kg (violet).

Supplementary Table 1: **Antibodies used for Immunohistochemistry (IHC)**

**Primary Antibodies**	**Company**	**Dilution**
rabbit anti-P-selectin (LS-B3578/57409)	LSBio	1:500
rat anti-CD31 (550274)	BD Biosciences	1:500
rat anti-CD34 (553731)	BD Biosciences	1:500
mouse anti-human CD62 (P-selectin)	LSBio	1:50
**Secondary Antibodies**	**Company**	**Dilution**
Donkey anti-rat (Secondary Antibody, Alexa Fluor 488)	Invitrogen	1:1000
Donkey anti-rabbit (Secondary Antibody, Alexa Fluor 647)	Invitrogen	1:1000

Supplementary Table 2: **Antibodies used for Western Blot (WB)**

**Primary Antibodies**	**Company**	**Dilution**
rabbit anti-P-selectin (LS-B3578/57409)	LSBio	1:1000
rabbit anti-P53 (CM5)	Leica	1:2000
rabbit anti-GAPDH (D16H11)	Cell Signaling	1:2000
**Secondary Antibody**	**Company**	**Dilution**
IRDye® 800RD Goat anti-Rabbit IgM	LI-COR	1:10000

Supplementary Table 3: **Sequence information for RT-qPCR**

Gene	Forward Primer (5'→ 3')	Reverse Primer (5'→ 3')
mGli1	CTTGTGGTGGAGTCATTGGA	GAGGTTGGGATGAAGAAGCA
mCav-1	GCGACCCCAAGCATCTCA	ATGCCGTCGAAACTGTGTGT
mGAPDH	CGTCCCGTAGACAAAATGGT	TCAATGAAGGGGTCGTTGAT
mP-selectin	CTATACCTGCTCCTGCTACCCAGGC	TTCACTCCACTGACCAGAGCCAGTG

Supplementary Table 4: **Sequence Information for CRISPR/Cas9 mediated knockout of *CAV1***

Target	ID	Sequence 1 - Forward* (5'→ 3')	Sequence 2 - Reverse* (5'→ 3')
Cav-1	sg1	CAAGCATCTCAACGACGACG	CGTCGTCGTTGAGATGCTTG
	sg2	ATGTGATTGCAGAACCAGAA	TTCTGGTTCTGCAATCACAT
GFP	sgGFP	CACCGGGGCGAGGAGCTGTTCACCG	AAACCGGTGAACAGCTCCTCGCCCC

*Sequences ordered as oligonucleotide duplexes from IDT (Integrated DNA Technologies).

Supplementary Table 5: shRNA hairpin sequence info for caveolin-1 (CAV1) and clathrin heavy chain (CLTC)

TRC Clone ID*	Gene	Species	Entrez ID	NCBI Ref Seq	Sequence
TRCN0000112662	CAV1	Mouse	12389	NM_007616	CGACGTGGTCAAGATTGACTT
TRCN0000112664	CAV1	Mouse	12389	NM_007616	GCTTCCTGATTGAGATTCAGT
TRCN0000112661	CAV1	Mouse	12389	NM_007616	CCGCTTGTTGTCTACGATCTT
TRCN0000379998	CLTC	Mouse	67300	NM_001003908	GATTACCAAGTATGGTTATAT
TRCN0000309447	CLTC	Mouse	67300	NM_001003908	CCAGAGAGATTTCTTCGTGAA
TRCN0000309523	CLTC	Mouse	67300	NM_001003908	GCCGACAAAGACAACACTAAT

*From The RNAi Consortium (TRC) lentiviral shRNA library. Each sequence was cloned into the pLKO.1 lentiviral vector to confer stable knockdown of indicated gene after transduction and subsequent selection.
