# Supplementary material for: P-selectin-targeted nanocarriers induce active crossing of the blood–brain barrier via caveolin-1-dependent transcytosis
Source: Nat Mater. 2023 Mar 2;22(3):391–9. doi: 10.1038/s41563-023-01481-9 (PMC9981459; doi:10.1038/s41563-023-01481-9)
Supplement: Supplementary file 2 — Reporting Summary [file 41563_2023_1481_MOESM2_ESM.pdf]

Corresponding author(s): Dr. Praveen Raju & Dr. Daniel A. Heller

Last updated by author(s): Jan 17, 2023

Reporting Summary

Nature Portfolio wishes to improve the reproducibility of the work that we publish. This form provides structure for consistency and transparency in reporting. For further information on Nature Portfolio policies, see our Editorial Policies and the Editorial Policy Checklist.

Statistics

For all statistical analyses, confirm that the following items are present in the figure legend, table legend, main text, or Methods section.

n/a	Confirmed	
☐	☒	The exact sample size (n) for each experimental group/condition, given as a discrete number and unit of measurement
☐	☒	A statement on whether measurements were taken from distinct samples or whether the same sample was measured repeatedly
☐	☒	The statistical test(s) used AND whether they are one- or two-sided<br>*Only common tests should be described solely by name; describe more complex techniques in the Methods section.*
☒	☐	A description of all covariates tested
☐	☒	A description of any assumptions or corrections, such as tests of normality and adjustment for multiple comparisons
☐	☒	A full description of the statistical parameters including central tendency (e.g. means) or other basic estimates (e.g. regression coefficient) AND variation (e.g. standard deviation) or associated estimates of uncertainty (e.g. confidence intervals)
☐	☒	For null hypothesis testing, the test statistic (e.g. F, t, r) with confidence intervals, effect sizes, degrees of freedom and P value noted<br>*Give P values as exact values whenever suitable.*
☒	☐	For Bayesian analysis, information on the choice of priors and Markov chain Monte Carlo settings
☒	☐	For hierarchical and complex designs, identification of the appropriate level for tests and full reporting of outcomes
☐	☒	Estimates of effect sizes (e.g. Cohen's d, Pearson's r), indicating how they were calculated

Our web collection on statistics for biologists contains articles on many of the points above.

Software and code

Policy information about availability of computer code

Data collection	No software was used for data collection.
Data analysis	GraphPad Prism(v.9.1.0) software was used for statistical analysis, QuPath (v.0.1.3) quantitative pathology & bioimage analysis software was used for quantification of fluorescence in histology samples. FCS Express (v.7.06) or FlowJo (10.6.1) was used for analysis of flow cytometry data.

For manuscripts utilizing custom algorithms or software that are central to the research but not yet described in published literature, software must be made available to editors and reviewers. We strongly encourage code deposition in a community repository (e.g. GitHub). See the Nature Portfolio guidelines for submitting code & software for further information.

Data

Policy information about availability of data

All manuscripts must include a data availability statement. This statement should provide the following information, where applicable:

- Accession codes, unique identifiers, or web links for publicly available datasets
- A description of any restrictions on data availability
- For clinical datasets or third party data, please ensure that the statement adheres to our policy

All referenced data is available in the manuscript or the supplementary materials. Additional data that support the findings of this study can be made available from the corresponding authors upon reasonable request.

Field-specific reporting

Please select the one below that is the best fit for your research. If you are not sure, read the appropriate sections before making your selection.

☒ Life sciences ☐ Behavioural & social sciences ☐ Ecological, evolutionary & environmental sciences

For a reference copy of the document with all sections, see nature.com/documents/nr-reporting-summary-flat.pdf

Life sciences study design

All studies must disclose on these points even when the disclosure is negative.

Sample size	Sample sizes were chosen based on previous literature in SHH-medulloblastoma tumor biology, nanomedicine, and our own expertise (PMID 31043743, PMID 25499213, PMID 22884371, PMID 27358497). In consultation with our biostatistics collaborator, our scientific approach incorporated explicit considerations of caveats of experimental models, including appropriate control groups and variables to ensure robustness of results.
Data exclusions	No data was excluded.
Replication	All in vivo experiments were performed in a minimum of n=3 to validate the results for each treatment group. Similarly, for in vitro studies, n=3 biologically independent replicates were used. We found this sample size is sufficient to control for any technical variations and extensive experience has shown to be sufficient to determine reproducible results from cultured cells.
Randomization	Assignment of sick mice to a treatment group was random. Since in vivo experiments addressed sex as a biological variable, both male and female mice were included in all mouse studies. In addition, as with studies involving mice, allocation of experimental groups for in vitro studies was random.
Blinding	Analysis of micro-CT data was performed by an investigator blinded to the treatment of the animals under analysis. Investigators were not blind to treatment groups for survival studies. The SHH medulloblastoma GEM model used in our studies has a reproducible phenotype (mean latency ~13.5 weeks and penetrance of ~85%). As with most GEM SHH medulloblastoma models, the rapid growth rate for the model used in our studies (characterized in PMID 25499213) results in mice presenting with obvious clinical symptoms (domed head, ataxia, weight loss) within the last week of life and was the timepoint used for initiation of treatment within the respective groups for survival studies. The rapid rate of symptom onset in mice within this narrow window of time and the need to prepare fucoidan-encapsulated vismodegib freshly to maintain nanoparticle stability precluded investigators from being blinded to treatment groups in the survival studies.

Reporting for specific materials, systems and methods

We require information from authors about some types of materials, experimental systems and methods used in many studies. Here, indicate whether each material, system or method listed is relevant to your study. If you are not sure if a list item applies to your research, read the appropriate section before selecting a response.

Materials & experimental systems

n/a	Involved in the study	
☐	☒	Antibodies
☐	☒	Eukaryotic cell lines
☒	☐	Palaeontology and archaeology
☐	☒	Animals and other organisms
☐	☒	Human research participants
☒	☐	Clinical data
☒	☐	Dual use research of concern

Methods

n/a	Involved in the study	
☒	☐	ChIP-seq
☐	☒	Flow cytometry
☒	☐	MRI-based neuroimaging

Antibodies

Antibodies used

Antibody (Catalog number/description); Provider; Dilution used.
rabbit anti-P-selectin (LS-B3578/57409); LSBio; 1 to 500.
rat anti-CD31 (550274); BD Biosciences; 1 to 500.
rat anti-CD34 (553731); BD Biosciences; 1 to 500.
mouse anti-human CD62P (LS-C44840); LSBio; 1 to 50.
Donkey anti-rat Secondary Antibody, Alexa Fluor 488 (A21208); Invitrogen; 1 to 1000.
Donkey anti-rabbit Secondary Antibody, Alexa Fluor 647 (A31573); Invitrogen; 1 to 1000
rabbit anti-P-selectin (LS-B3578/57409); LSBio; 1 to 1000.
rabbit anti-P53 (CM5); Leica; 1 to 2000.
rabbit anti-GAPDH (D16H11); Cell Signaling; 1 to 2000.
IRDye® 800RD Goat anti-Rabbit IgM (P/N 926-32232); LI-COR; 1 to 10000.

Validation	In addition to the validation of primary antibodies as described on the manufacturer's websites, we further validated our data provided in the manuscript using secondary antibody-only controls and blocking peptide studies when available. In addition, we utilized bEnd.3 brain endothelial cell lines that were genetically knocked out for SELP or Cav1 using Crispr-Cas9 approaches as well as mouse SELP null and Cav1 null alleles to further confirm the specificity of these antibodies as shown in the manuscript. Finally, the focal nature of P-selectin expression in brain tumor endothelium including following ionizing radiation and not in normal brain endothelium (previously described in PMID 9823335) also allowed us to use the normal brain regions of the same animals as internal controls for the specificity of our antibody staining.

Eukaryotic cell lines

Policy information about cell lines

Cell line source(s)	bEnd.3 cells were purchased from ATCC. Cav1 knockout bEnd.3 cells were generated by Jacob Boyer (Rosen Lab, MSKCC) LentiX cells were generously gifted by Kristen Vogt (Scheinberg Lab, MSKCC).
Authentication	Cell lines were not externally authenticated.
Mycoplasma contamination	Cell lines routinely tested negative for mycoplasma contamination.
Commonly misidentified lines (See ICLAC register)	No cell lines used in this study were found in the database of commonly misidentified cell lines that is maintained by ICLAC

Animals and other organisms

Policy information about studies involving animals; ARRIVE guidelines recommended for reporting animal research

Laboratory animals	SHH-MB mice (Ptf1acre/+;Ptch1fl/fl) were generated by intercrossing Ptf1acre/+ mice with Ptch1fl/fl mice and maintained on a C57BL/6 background. Cav1 null (JAX Stock No:007083) and Selp null mice (JAX Stock No: 008432) were bred with SHH-MB mice to generate Ptf1acre/+;Ptch1fl/fl;Cav1-/- and Ptf1acre/+;Ptch1fl/fl;Selp-/- SHH-MB mice, respectively. The genotype of each mouse was confirmed by PCR genotyping of tail biopsy using primers for Ptch1, Cre, Cav1, and Selp (see Table S3 for primer sequences). Treatment studies were performed in advanced stage symptomatic medulloblastoma mice between 12-20 weeks age. Both sexes were used for all studies. Animals were housed on a 12-hour (hr) light/dark cycle with standard mouse room temperatures between ~18-23 C with ~40-60% humidity per MSKCC and Weill Cornell RARC animal facility guidelines and were given access to food and water ad libitum.
Wild animals	This study did not involve wild animals.
Field-collected samples	This study did not involve samples collected from the field.
Ethics oversight	All mice in this study were maintained under protocols approved by the Institutional Animal Care and Use Committee at Weill Cornell Medicine and Memorial Sloan Kettering Cancer Center.

Note that full information on the approval of the study protocol must also be provided in the manuscript.

Human research participants

Policy information about studies involving human research participants

Population characteristics	This study involves staining of de-identified histological slides of brain tumor samples from patients with medulloblastoma seen at Nationwide Children's Hospital and NYU Langone Medical Center according to ethical guidelines and approved institutional IRB protocols.
Recruitment	No recruitment was performed for this study.
Ethics oversight	Approved by institutional IRB protocols at Nationwide Children's Hospital and NYU Langone Medical Center.

Note that full information on the approval of the study protocol must also be provided in the manuscript.

Flow Cytometry

Plots

Confirm that:

☒ The axis labels state the marker and fluorochrome used (e.g. CD4-FITC).

☒ The axis scales are clearly visible. Include numbers along axes only for bottom left plot of group (a 'group' is an analysis of identical markers).

☒ All plots are contour plots with outliers or pseudocolor plots.

☒ A numerical value for number of cells or percentage (with statistics) is provided.

Methodology

Sample preparation	Murine brain endothelial (bEnd.3) cells were plated in a 12-well plate at a density of 150,000 cells/well in 1mL of media (DMEM, 10% FBS, 1% P/S). Murine brain endothelial (bEnd.3) cells were plated in a 12-well plate at a density of 150,000 cells/well in 1mL of media (DMEM, 10% FBS, 1% P/S). Once confluent, cells in treatment groups receiving ionizing radiation were exposed to 0.25 Gy XRT. After 1 hour, cells were collected, transferred to microcentrifuge tubes, and fixed on ice using 2% paraformaldehyde. Fixed cells were washed twice with PBS and then resuspended in 100 µL FACS buffer (PBS with 2% FBS). Cells were stained with 2 µl of anti-P-selectin antibody (Biolegend, Cat #148310) and incubated at room temperature for 30 minutes. Cells were then washed twice with PBS, resuspended in 300µl of FACS buffer, and transferred to FACS tubes for analysis.). To assess nanoparticle uptake, cells were incubated with nanoparticles for 30 minutes at 37°C. Afterwards, cells were washed twice with PBS and resuspended in freshly prepared FACS buffer containing propidium iodide as a viability stain. Data was collected on a BD LSR II flow cytometer, using the APC-Cy7 channel (excitation with 633 nm red laser, detection with 780/60nm bandpass filter) to detect fluorescent signal from the IR-dyes within the nanoparticles.
Instrument	Data was collected on a BD LSR II flow cytometer
Software	Data was analyzed using FCS Express (v.7.06) or FlowJo (10.6.1) software.
Cell population abundance	The SHH medulloblastoma GEM model used in these studies is similar to most murine medulloblastoma models in that the tumors are essentially represented by proliferating tumor cells with relatively few other cell type in the microenvironment. Furthermore, these neuroepithelial tumor cells are relatively uniform in shape and with a paucity of genetic mutations as shown by several publications in the medulloblastoma field. In addition, we have characterized our Ptf1acre/+; Ptch1fl/fl GEM model to have ~95-97% tumor cells with ~5-7% tumor microenvironment cells that are predominantly tumor-associated macrophages similar to human SHH medulloblastoma.<br><br>Importantly, we utilized advanced stage medulloblastoma tumors for our treatment studies and previously showed in PMID 25499213 that the tumors at these advanced symptomatic stages represented a significant tumor volume within the cerebellum of these mice. We analyzed the effects of fucoidan-encapsulated vismodegib nanoparticles on tumor bulk, i.e., mostly proliferative tumor cells, at these advanced stages in replicative samples using qPCR for Gli1 target inhibition using GAPDH as in internal control. Furthermore, in our survival studies we compared control treated mice that typically succumb to disease within one week due to this large tumor volume and showed efficacy with fucoidan-encapsulated vismodegib through its effects on this large bulk population therefore further minimizing the applicability of absolute cell population abundance to this study.
Gating strategy	For all flow cytometry experiments cells were first identified by FSC/SSC such that all cells were visible on the plot. Positive populations were determined by comparing experimental samples against unstained controls. Example of gating strategy is provided in the Supplementary Information file attached.

☒ Tick this box to confirm that a figure exemplifying the gating strategy is provided in the Supplementary Information.
