# Supplementary material for: P-selectin-targeted nanocarriers induce active crossing of the blood–brain barrier via caveolin-1-dependent transcytosis
Source: Nat Mater. 2023 Mar 2;22(3):391–9. doi: 10.1038/s41563-023-01481-9 (PMC9981459; doi:10.1038/s41563-023-01481-9)
Supplement: Supplementary file 6 — Unprocessed immunoblot image for Supplementary Fig. 2b. [file 41563_2023_1481_MOESM6_ESM.pdf]

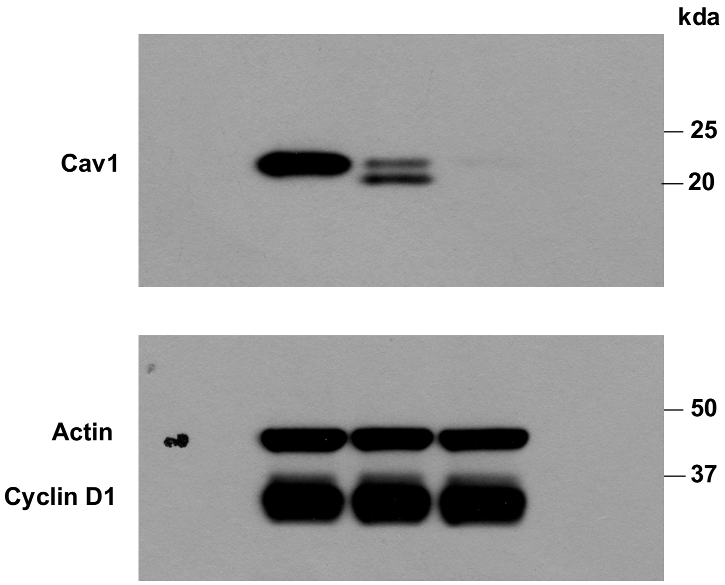


**Source Data for Supplementary Data Fig. 2.** Unprocessed image of western blot for caveolin-1, actin, and cyclin d1.
