# Supplementary material for: P-selectin-targeted nanocarriers induce active crossing of the blood–brain barrier via caveolin-1-dependent transcytosis
Source: Nat Mater. 2023 Mar 2;22(3):391–9. doi: 10.1038/s41563-023-01481-9 (PMC9981459; doi:10.1038/s41563-023-01481-9)
Supplement: Source Data Extended Data Fig. 3 — Unprocessed imunoblot images. [file 41563_2023_1481_MOESM24_ESM.pdf]

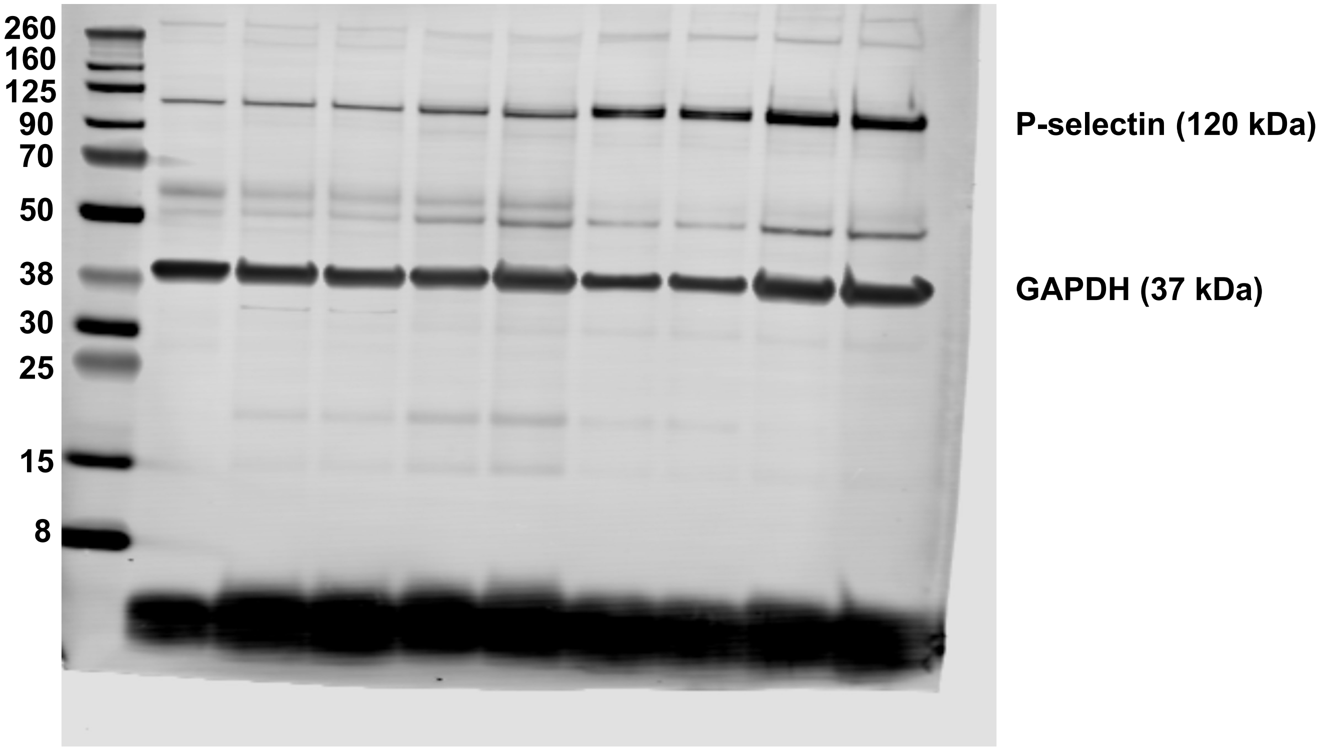

Source Data for Extended Data Fig. 3. Unprocessed image of western blot for P-selectin and GAPDH

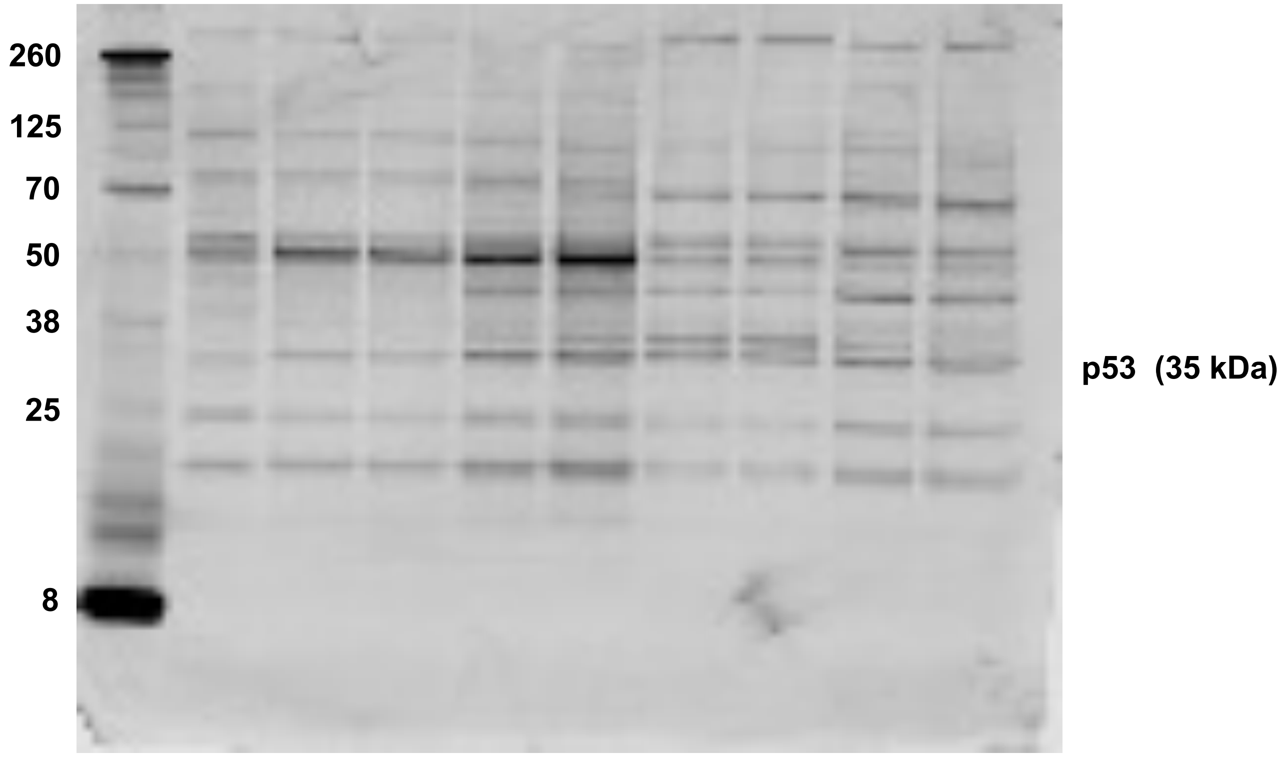

Source Data for Extended Data Fig. 3. Unprocessed image of western blot for p53
